# The mechanosensitive lncRNA *Neat1* promotes osteoblast function through paraspeckle-dependent *Smurf1* mRNA retention

**DOI:** 10.1038/s41413-022-00191-3

**Published:** 2022-02-24

**Authors:** Caizhi Liu, Xingcheng Gao, Yuheng Li, Weijia Sun, Youjia Xu, Yingjun Tan, Ruikai Du, Guohui Zhong, Dingsheng Zhao, Zizhong Liu, Xiaoyan Jin, Yinlong Zhao, Yinbo Wang, Xinxin Yuan, Junjie Pan, Guodong Yuan, Youyou Li, Wenjuan Xing, Guanghan Kan, Yanqing Wang, Qi Li, Xuan Han, Jianwei Li, Shukuan Ling, Yingxian Li

**Affiliations:** 1 State Key Laboratory of Space Medicine Fundamentals and Application, China Astronaut Research and Training Center, Beijing, China; 2 The Key Laboratory of Aerospace Medicine, Ministry of Education, The Fourth Military Medical University, Xi’an, Shaanxi China; 3 The Second Affiliated Hospital of Soochow University, Suzhou, Jiangsu China; 4 College of Life Sciences, Hebei Normal University, Shijiazhuang, Hebei China; 5 Medical College of Soochow University, Suzhou, Jiangsu China; 6 Medical School of Southeast University, Nanjing, Jiangsu China

**Keywords:** Bone, Bone quality and biomechanics

## Abstract

Mechanical stimulation plays an important role in bone remodeling. Exercise-induced mechanical loading enhances bone strength, whereas mechanical unloading leads to bone loss. Increasing evidence has demonstrated that long noncoding RNAs (lncRNAs) play key roles in diverse biological, physiological and pathological contexts. However, the roles of lncRNAs in mechanotransduction and their relationships with bone formation remain unknown. In this study, we screened mechanosensing lncRNAs in osteoblasts and identified *Neat1*, the most clearly decreased lncRNA under simulated microgravity. Of note, not only *Neat1* expression but also the specific paraspeckle structure formed by *Neat1* was sensitive to different mechanical stimulations, which were closely associated with osteoblast function. Paraspeckles exhibited small punctate aggregates under simulated microgravity and elongated prolate or larger irregular structures under mechanical loading. *Neat1* knockout mice displayed disrupted bone formation, impaired bone structure and strength, and reduced bone mass. *Neat1* deficiency in osteoblasts reduced the response of osteoblasts to mechanical stimulation. In vivo, *Neat1* knockout in mice weakened the bone phenotypes in response to mechanical loading and hindlimb unloading stimulation. Mechanistically, paraspeckles promoted nuclear retention of E3 ubiquitin ligase *Smurf1* mRNA and downregulation of their translation, thus inhibiting ubiquitination-mediated degradation of the osteoblast master transcription factor Runx2, a Smurf1 target. Our study revealed that *Neat1* plays an essential role in osteoblast function under mechanical stimulation, which provides a paradigm for the function of the lncRNA-assembled structure in response to mechanical stimulation and offers a therapeutic strategy for long-term spaceflight- or bedrest-induced bone loss and age-related osteoporosis.

## Introduction

Bone is a dynamic tissue that endures constant remodeling throughout life.^1^ Mechanical loading is important for maintaining bone homeostasis. This process involves osteoblast-governed bone formation and osteoclast-induced bone resorption.^2,3^ Weightlessness-induced bone loss continues during long-term spaceflight.^4^ This issue has become one of the most important factors limiting long-term orbital spaceflight. Osteoblast lineage cells can perceive and orchestrate the modeling and remodeling response to mechanical signals.^5,6^ However, the mechanisms by which these cells sense and transduce mechanical stimuli derived from loading or unloading are poorly understood.

Long noncoding RNAs (lncRNAs) are transcripts of more than 200 nucleotides in length^7^ that function as regulators at the transcriptional, translational, and post-translational levels.^8^ By interacting with DNA, RNA, or protein, lncRNAs are involved in many cellular processes, including gene regulation^9,10^ and the formation and regulation of organelles and nuclear condensates.^11,12^ However, their roles in mechanical signal transduction remain unknown.

The lncRNA nuclear paraspeckle assembly transcript 1 (*Neat1*, nuclear-enriched abundant transcript 1), which forms the backbone of subnuclear ‘paraspeckle’ bodies, has been shown to be involved in many fundamental cellular functions.^11,13^ *Neat1* is transcribed from the multiple endocrine neoplasia (*Men*) locus on chromosome 19 in mice. *Neat1_1* (3.2 kb, also known as *Menε*) and *Neat1_2* (20 kb, also known as *Menβ*) are two isoforms produced through alternate 3′-end processing. *Neat1_1* overlaps completely at the 5′ end of *Neat1_2*. *Neat1_2* was demonstrated to be a potent RNA component for paraspeckle construction.^11,14,15^ *Neat1_2*, in combination with many binding proteins, including NONO (p54nrb), PSF (SFPQ) and FUS, plays an architectural role in the stabilization and assembly of paraspeckles.^16,17^ In the paraspeckle, the 5′ region and 3′ end of *Neat1_2* are located at the paraspeckle periphery, and the middle region is located in the interior.^16^

Paraspeckles are highly organized and dynamically regulated membraneless nuclear bodies^18^ that respond to a variety of basic physiological processes, including stress responses,^13,19^ metabolic condition alterations^19^, and cellular differentiation,^11^ as well as biomechanical changes in cancer cells.^20^ Paraspeckles are capable of retaining certain RNA species in the nucleus, sequestering specific component proteins and are involved in transcription and miRNA processing.^21^ The 3′-UTRs of many mRNAs contain Alu elements or short interspersed nuclear elements (SINEs), which can promote A-to-I hyperediting. Hyperedited mRNAs can be retained in the nucleus by binding with the paraspeckle proteins NONO and PSF.^22,23^ *Neat1* is also involved in the targeting of RNAs through direct RNA-RNA interactions through its 5′ end.^21^ Disassembly of paraspeckles leads to various abnormalities, including fibroblast death,^24,25^ early developmental arrest,^26^ decreased fertility and mammary dysplasia.^27,28^ However, its roles in osteoblast function have not been determined.

In this study, we revealed the essential role of *Neat1* and paraspeckles in osteoblast function. We found that the *Neat1* expression level and paraspeckle morphology are sensitive to mechanical stimuli, including simulated microgravity (MG), hypergravity, fluid shear stress (FSS), and stiff matrix stimulation, which are closely associated with osteoblast function. Genetic knockout of *Neat1* inhibited osteoblast differentiation in vitro and bone formation in vivo. *Neat1*-deficient osteoblasts lost their response to mechanical stimulation, and the bone tissue of *Neat1* knockout (*Neat1*-KO) mice was insensitive to mechanical loading or hindlimb unloading. The underlying mechanism was mainly through sequestering E3 ubiquitin ligase *Smurf1* mRNA in paraspeckles, thereby reducing the Smurf1 (Smad ubiquitin regulatory factor 1) protein level and inhibiting the proteasomal degradation of Runx2, a master transcription factor in osteogenesis. Taken together, our study revealed a new mechanism in which paraspeckles mediated osteoblast function and bone formation through nuclear retention of an E3 ubiquitin ligase, which provided a potential therapeutic strategy against osteoporosis.

## Results

### Mechanosensitive lncRNA screening and paraspeckle changes in osteoblasts under different mechanical stimuli

**Fig. 1:**
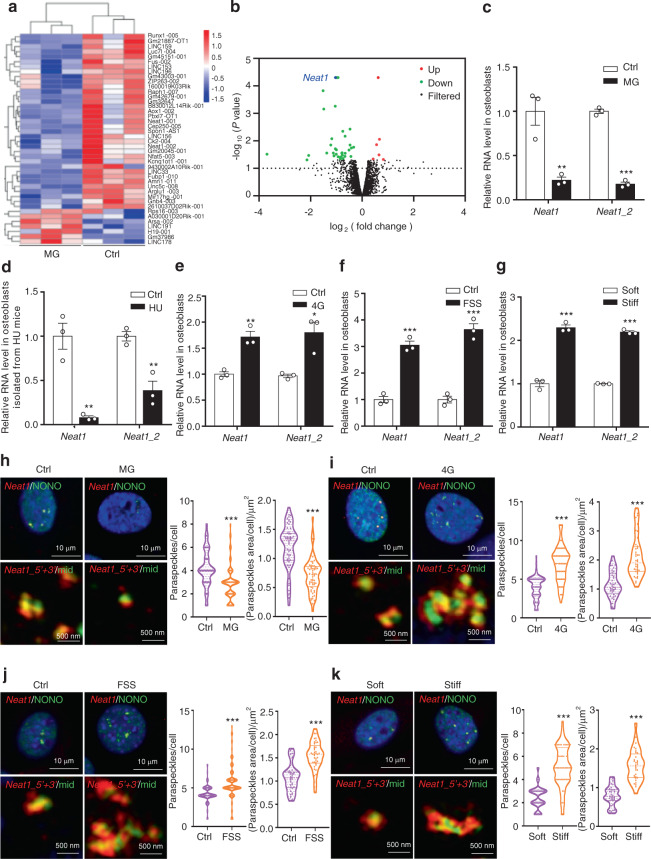
Mechanosensitive lncRNA screening and paraspeckle changes in osteoblasts under different mechanical stimulations. **a** Heatmap of differentially expressed profiles of lncRNAs between the control and simulated microgravity (MG)-exposed mouse primary osteoblasts, as revealed by RNA-Seq with a cutoff fold change >1.3. *n* = 3 in each group. **b** Volcano plots indicating the altered lncRNAs in the control and MG-exposed mouse primary osteoblasts. **c** The expression of *Neat1* and *Neat1_2* in the control and MG-exposed osteoblasts. *n* = 3 in each group. **d** Analysis of total *Neat1* and *Neat1_2* expression in osteoblasts isolated from the control (Ctrl) mice and mice with hindlimb unloading (HU). **e** The expression of total *Neat1* and *Neat1_2* in the control and 4G mouse primary osteoblasts. f. Analysis of total *Neat1* and *Neat1_2* expression in the mouse primary osteoblasts treated with fluid flow shear stress (FSS). The data from **d**–**f** are presented as the mean ± s.e.m. from three independent experiments. **g** The expression of total *Neat1* and *Neat1*-2 in mouse primary osteoblasts cultured on soft or stiff matrix. The results are presented as the mean ± s.e.m. from three independent experiments. **h** Representative images of paraspeckles in the control and MG-exposed mouse primary osteoblasts under confocal microscopy (*n* = 83 and 96 cells in each group) and SIM microscopy (*n* = 56 and 60 cells in each group). **i** Representative images of paraspeckles in the control and 4G mouse primary osteoblasts under confocal microscopy (*n* = 71 and 83 cells in each group) and SIM microscopy (*n* = 37 and 40 cells in each group). **j** Representative images showing paraspeckles in mouse primary osteoblasts under confocal microscopy (*n* = 107 and 127 cells in each group) and SIM microscopy (*n* = 34 and 41 cells in each group) after FSS treatment. **k** Representative images showing paraspeckles in the mouse primary osteoblasts cultured on soft and stiff matrix under confocal microscopy (*n* = 52 and 60 cells in each group) and SIM microscopy (*n* = 31 and 32 cells in each group). Paraspeckle quantification was performed and graphed to the right of each representative image. The experiments of **h**–**k** were repeated more than three times. *P < 0.05, **P < 0.01, ***P < 0.001

Unloading-induced bone loss mainly results from reduced mechanical signals to osteoblasts. To identify the mechanosensitive lncRNAs in osteoblasts, we performed RNA sequencing of mouse primary osteoblasts exposed to simulated MG. We examined the capacity of osteoblast differentiation under this condition. The osteogenic marker genes of osteoblast differentiation include alkaline phosphatase (ALP), bone gamma-carboxyglutamate protein (Bglap) and collagen type I alpha (Col1α1). The levels of these three mRNAs were downregulated, and the ALP activity was clearly decreased under MG conditions (Fig. S1a–c). The differentially expressed lncRNAs (fold change ≥ 1.3 was used as the quantification threshold) between the control and MG mouse primary osteoblasts are shown in heatmaps (Fig. 1a). A total of 42 lncRNAs displayed distinct expression patterns in osteoblasts under MG conditions, including 7 lncRNAs with upregulated and 35 with downregulated expression (Fig. 1b). We further verified 2 lncRNAs with upregulated and 12 with downregulated expression by quantitative reverse transcriptase-PCR. LINC178 and LINC191 were clearly increased in the MG-exposed osteoblasts compared with the controls (Fig. S1d). Among the lncRNAs with downregulated expression, *Neat1* showed the most significant reduction (Fig. 1c, S1e). To further confirm that *Neat1* changed in vivo under MG conditions, we isolated osteoblasts from the bone of the mice that underwent hindlimb unloading, which is a mechanical unloading animal model. Consistent with this model, in isolated osteoblasts, the *Neat1* and *Neat1_2* expression levels were significantly downregulated compared with the controls (Fig. 1d). These findings suggested that downregulated *Neat1* expression is associated with decreased osteoblast function under mechanical unloading conditions.

To further confirm the response of *Neat1* expression to diverse mechanical loads in osteoblasts, we determined the changes in *Neat1* under the conditions of 4G hypergravity, FSS, and stiff matrix stimulation. The levels of osteogenic marker genes were upregulated, and ALP activity was substantially increased after mechanical loading application (Fig. S1f–n). Accordingly, the *Neat1* and *Neat1_2* levels were also significantly increased under these mechanical loading conditions (Fig. 1e–g). These results indicated that *Neat1* is involved in the osteoblast response to mechanical stimulation.

*Neat1* is an indispensable structural component of paraspeckles, which are dynamic in their response to various forms of stress, including different mechanical stimulation responses. Therefore, to test for the existence of *Neat1* in the form of a paraspeckle in the nucleus, we performed nuclear and cytoplasmic fraction isolation and RNA fluorescence in situ hybridization (FISH) in osteoblasts. *Neat1* was predominantly localized in the nucleus in osteoblasts (Fig. S2a, b). We observed *Neat1* foci in osteoblasts using RNA probes that recognized either the region common to both *Neat1* isoforms or the region specific to *Neat1_2*. As described in a previous study, *Neat1* puncta appeared as core shell spheroidal structures, with the 5′ and 3′ regions of *Neat1* located around the middle region (Fig. S2c). FISH followed by immunofluorescence with an antibody against the paraspeckle protein NONO demonstrated that NONO overlapped very well with the *Neat1* puncta, which further indicated the existence of paraspeckles in osteoblasts (Fig. S2d). Under simulated MG conditions, we observed that the number and total area of paraspeckles in osteoblasts were significantly decreased, and the morphology of the paraspeckles exhibited a smaller round-shaped structure compared with the controls (Fig. 1h). In contrast, compared with those of the control group, the number and total area of paraspeckles displayed a twofold increase in osteoblasts under 4G hypergravity conditions (Fig. 1i). Furthermore, the number and total area of paraspeckles in osteoblasts under FSS and stiff matrix stimuli were increased, as expected (Fig. 1j, k). Under mechanical loading conditions, numerous small round-shaped paraspeckles were aggregated and formed elongated prolate structures and irregular shapes of paraspeckles. Above all, these results demonstrated that *Neat1* and paraspeckles formed by acting as mechanical sensors in osteoblasts and displayed changes in expression levels and structures in response to different mechanical stimulations.

### Correlation of *Neat1* and paraspeckles with osteoblast function

**Fig. 2:**
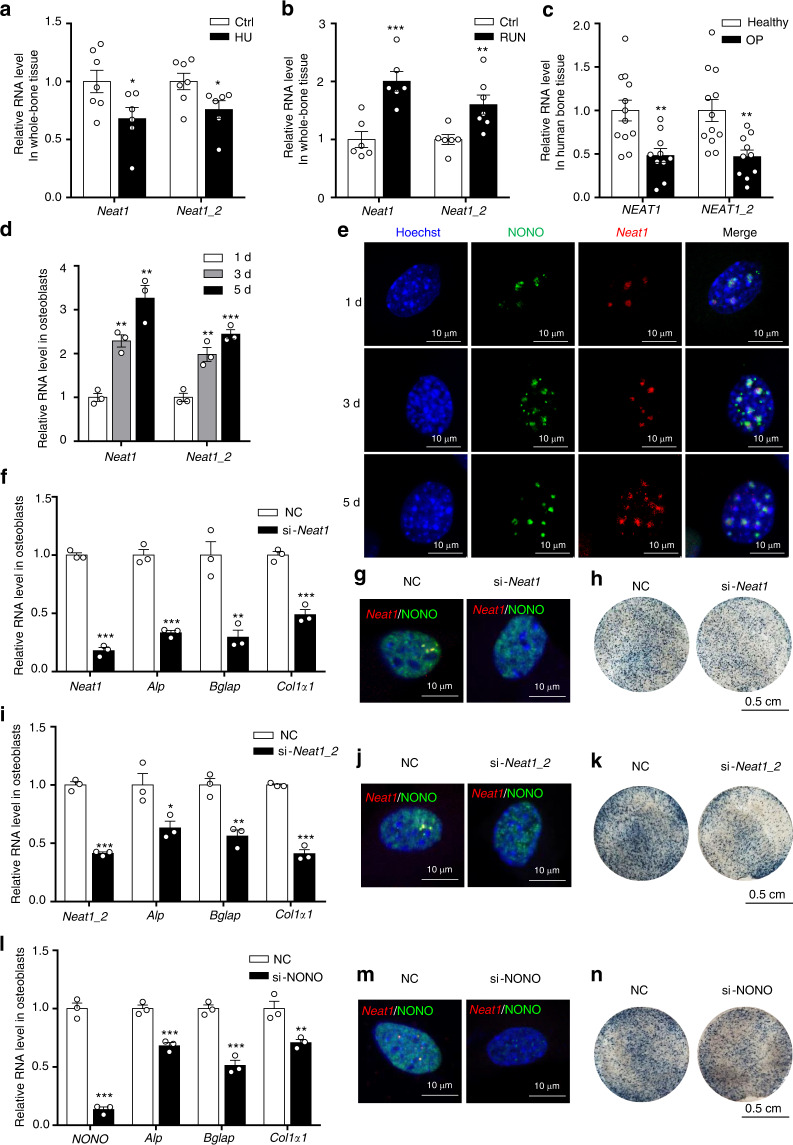
Osteoblast function is closely correlated with *Neat1* expression and paraspeckle formation. **a** Analysis of total *Neat1* and *Neat1_2* expression in whole-bone tissue from the control mice (*n* = 7) and mice with hindlimb unloading (*n* = 6). **b** Total *Neat1* and *Neat1_2* expression in whole-bone tissue from control (*n* = 6) and mechanically loaded (*n* = 6) mice. **c** The expression of total *NEAT1* and *NEAT1_2* in the bone tissue of healthy controls (*n* = 12) and patients with OP (*n* = 10). **d** The expression of total *Neat1* and *Neat1_2* in the process of osteoblast differentiation. The experiment was repeated for more than three times. **e** Representative images of confocal observations of paraspeckles during osteoblast differentiation. **f** Analysis of the expression of the osteogenic marker genes *Alp*, *Bglap* and *Col1α1* after *Neat1* knockdown. Representative results of three independent experiments are shown. **g** Representative FISH images of paraspeckles in osteoblasts upon *Neat1* knockdown. **h** Analysis of ALP staining in primary osteoblasts after *Neat1* knockdown. **i** Expression of the osteogenic marker genes *Alp*, *Bglap* and *Col1α1* after *Neat1_2* knockdown. The experiment was repeated for more than three times. **j** Representative FISH images of paraspeckles in osteoblasts upon *Neat1_2* knockdown. **k** Representative ALP staining images of the control and *Neat1_2* knockdown osteoblasts. **l** Analysis of the expression of the osteogenic marker genes *Alp*, *Bglap*, and *Col1α1* after paraspeckle NONO knockdown. Representative results of three independent experiments are shown. **m** Representative FISH images of paraspeckles in osteoblasts upon NONO knockdown. **n** Representative ALP staining images of control and *NONO* knockdown osteoblasts. Data represent three independent experiments. *P < 0.05, **P < 0.01, ***P < 0.001

To detect the potential role of *Neat1* and paraspeckles in osteoblast function, we determined the *Neat1* and *Neat1_2* expression levels in bone tissue from mice with hindlimb unloading/loading and patients with osteoporosis. We found that the total *Neat1* and *Neat1_2* expression levels were obviously decreased in bone tissue with unloading treatment (Fig. 2a). In contrast, their levels were significantly increased under loading treatment (Fig. 2b). In patients with clinical osteoporosis, total *NEAT1* and *NEAT1_2* levels in human bone specimens were substantially reduced compared with those in the controls without osteoporosis (Fig. 2c), and their levels were positively correlated with the T score in the samples of the patients with osteoporosis (Fig. S3a, b). These results demonstrated that the *Neat1* level is associated with osteoblast function under physiological or pathological conditions.

To identify the changes in *Neat1* expression and paraspeckle assembly during the process of osteoblast differentiation, we induced osteoblasts with osteogenic medium for 1, 3 or 5 days. The results showed that the *Neat1* and *Neat1_2* levels gradually increased during the course of osteoblastic differentiation (Fig. 2d), and the number of paraspeckles also accordingly increased (Fig. 2e, Fig. S3c), accompanied by elevated osteoblast master regulator Runx2 protein levels, osteogenic marker gene expression and Alp activity in osteoblasts (Fig. S3d–f). To further explore the role of paraspeckles in osteoblasts, we used siRNAs against *Neat1* and the paraspeckle component proteins *NONO* and *PSF* to disturb paraspeckle assembly. The knockdown of total *Neat1* or *Neat1_2* resulted in paraspeckle destruction, decreased osteogenic marker gene expression and repressed ALP activity (Fig. 2f–k). When NONO or PSF was knocked down, the paraspeckles disappeared, and the osteoblast activity was significantly reduced (Fig. 2l–n, Fig. S3g–i). Additionally, overexpression of the short isoform *Neat1_1* had no effect on the expression of osteoblastic marker genes or ALP activity (Fig. S3j, k). Taken together, these results indicated that *Neat1* and paraspeckles are closely correlated with osteoblast function.

### *Neat1* deficiency inhibits bone formation in vivo

**Fig. 3:**
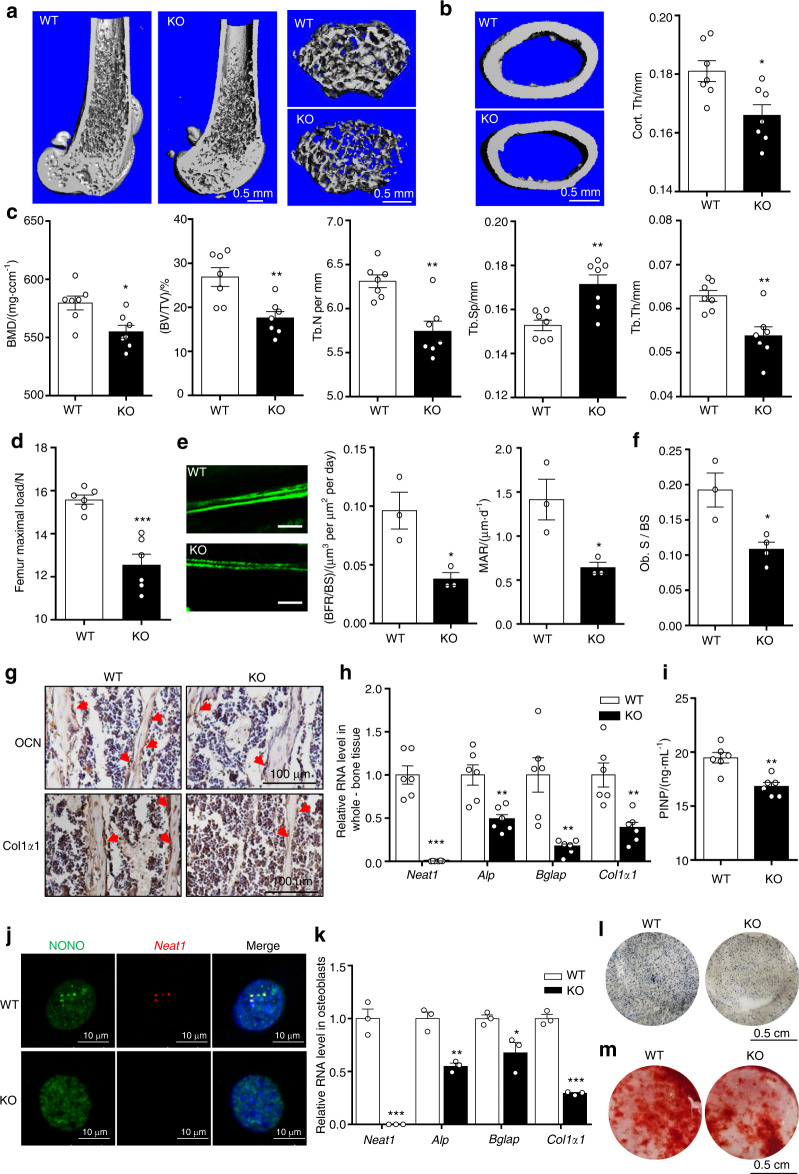
*Neat1* deficiency resulted in reduced bone formation and impaired osteoblast function. **a**, **b** Representative trabecular and cortical bone 3D μ-CT images from distal femurs of 2-month-old WT and *Neat1*-KO male mice. Scale bars, 0.5 mm. Cortical bone thickness of distal femurs from the WT (*n* = 7) and *Neat1*-KO (*n* = 7) mice was analyzed. **c** Quantitative µ-CT analysis of BMD, BV/TV, Tb.N, Tb.Sp and Tb.Th from the WT (*n* = 7) and *Neat1*-KO mice (*n* = 7). **d** Femur maximal load determined by three-point bending in the WT (*n* = 6) and *Neat1*-KO (*n* = 6) mice. **e** Double calcein labeling images showing new bone formation from the WT (*n* = 3) and *Neat1*-KO (*n* = 3) mice. BFR/BS, bone formation rate/bone surface. MAR, mineral apposition rate. Scale bars, 50 μm. **f** Histomorphometric analysis of osteoblast surface per bone surface in the WT and *Neat1*-KO mice. *n* = 3 in each group. **g** Immunohistochemical staining of OCN and Col1α1 in tibial sections from 6-week-old *Neat1*-KO (*n* = 3) mice and WT (*n* = 3) mice. Scale bars, 100 μm. **h** Analysis of osteogenic marker gene expression in bone tissue from the WT (*n* = 6) and *Neat1*-KO (*n* = 6) mice. **i** ELISA analysis of serum PINP (ng·mL^**−**1^) in 2-month-old WT (*n* = 6) and *Neat1*-KO (*n* = 6) mice. **j** Representative FISH images of paraspeckles in the WT and *Neat1*-KO osteoblasts. Scale bars, 10 μm. **k** Analysis of osteogenic marker genes in primary osteoblasts isolated from the WT and *Neat1*-KO mice. *n* = 3 in each group. **l** ALP staining images of the WT and *Neat1*-KO osteoblasts induced with osteogenic medium for 3 days. m. Representative Alizarin red staining images of the WT and *Neat1*-KO osteoblasts. *P < 0.05, **P < 0.01, ***P < 0.001

To determine the role of *Neat1* in regulating osteoblast function and bone formation in vivo, we investigated *Neat1* knockout (*Neat1*-KO) mice (Fig. S4a). Notably, compared with the wild-type (WT) control mice, the *Neat1*-KO mice displayed a shorter stature, smaller sternum, and delayed osteogenesis of long bones, which were indicated by Alizarin red and Alcian blue staining at postnatal day 2 (Fig. S4b, c). Furthermore, the *Neat1*-KO mice exhibited a smaller size, and their body weight decreased significantly compared with that of the control littermates (Fig. S4d, e). Micro-CT analysis of the trabecular architecture showed that the *Neat1*-KO mice displayed lower bone mass than the WT mice (Fig. 3a). The femoral cortical thickness was less than that in the WT mice (Fig. 3b). Detailed analysis of the skeletal phenotype revealed decreased bone mineral density (BMD), bone volume per tissue volume (BV/TV), trabecular number (Tb.N), and trabecular thickness (Tb.Th) and increased trabecular space (Tb.Sp) in the *Neat1*-KO mice (Fig. 3b, c). Consistently, the femoral strength of the *Neat1*-KO mice was greatly reduced compared with that of the WT mice (Fig. 3d). Moreover, labeling with calcein suggested that the *Neat1*-KO mice exhibited decreased bone formation and mineral appositional rates (Fig. 3e). In addition, the analysis of histomorphometric parameters revealed that the osteoblast surface/bone surface in tibias from the *Neat1*-KO mice was significantly decreased compared with that of the WT mice (Fig. 3f). Accordingly, the levels of the osteoblastic markers osteocalcin (OCN) and Col1α1 were greatly decreased in the *Neat1*-KO mice compared with those in the tibias of the WT mice (Fig. 3g, Fig. S4f, g). Quantitative reverse transcriptase-PCR (q-PCR) analysis showed that the expression of osteogenic marker genes was also significantly decreased in whole-bone tissue from the *Neat1*-KO mice (Fig. 3h). Consistent with the bone loss phenotype, the serum level of the bone formation marker procollagen type I N-terminal propeptide (PINP) was significantly decreased (Fig. 3i). Taken together, these findings suggested that *Neat1* knockout severely inhibits bone formation in vivo.

In addition, primary osteoblasts were isolated from the WT and *Neat1*-KO mice. In the WT osteoblasts, there were obvious signs of paraspeckles, as indicated by colocalization of *Neat1* with NONO. In the *Neat1*-KO osteoblasts, there was no distinct paraspeckle signal (Fig. 3j). The expression of osteogenic marker genes was significantly downregulated in the *Neat1*-KO osteoblasts (Fig. 3k). Furthermore, the *Neat1*-KO osteoblasts displayed greatly reduced ALP activity and delayed mineralization compared with the control osteoblasts (Fig. 3l, m). These data demonstrated that *Neat1* KO seriously impairs osteoblast function and bone formation.

### Paraspeckles mediated *Smurf1* mRNA nuclear retention in osteoblasts

**Fig. 4:**
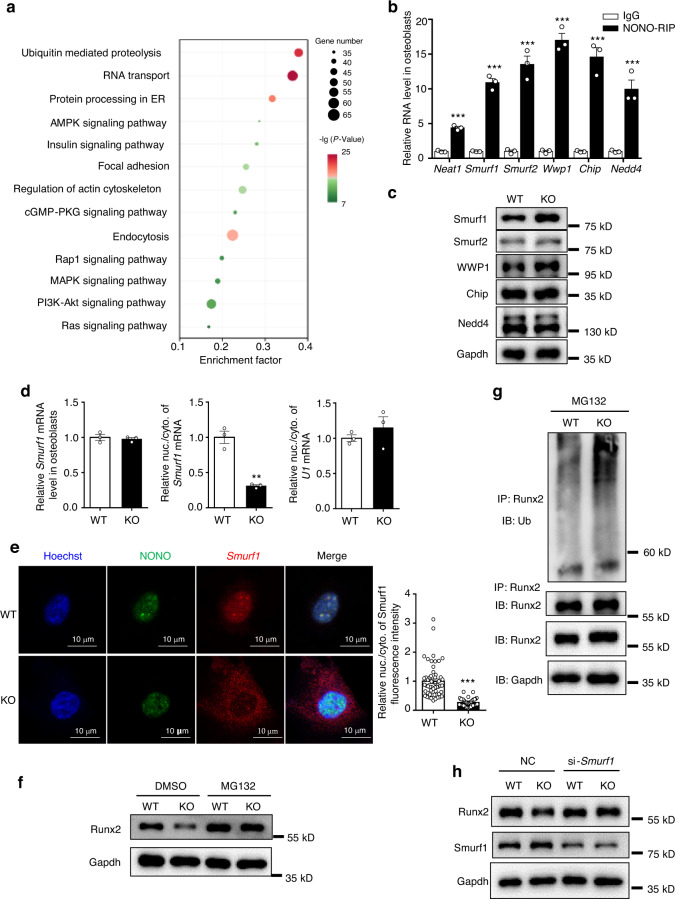
Identification of *Neat1*-interacting mRNA and the nuclear retention of *Smurf1* in osteoblasts. **a** KEGG signaling pathway enrichment analysis of genes in osteoblasts after RIP-Seq. *n* = 3 of each group. **b** Enhanced enrichment of candidate E3 ubiquitin ligase mRNA revealed by native NONO immunoprecipitation in osteoblasts. Data are presented as the mean ± s.e.m. from three independent experiments. **c** Protein levels of candidate E3 ubiquitin ligases were detected in the WT and *Neat1*-KO osteoblasts. The experiment was repeated for more than three times. **d** Quantitative PCR showed no changes in total *Smurf1* mRNA between the WT and *Neat1*-KO osteoblasts. Enhanced nucleocytoplasmic export of *Smurf1* mRNA was shown in *Neat1*-KO osteoblasts. *U1* was used as a negative control for the nuclear and cytoplasmic ratios. The results are presented as the mean ± s.e.m. from three independent experiments. **e** Representative image of *Smurf1* RNA FISH in the WT and *Neat1*-KO osteoblasts. The quantitative data of *Smurf1* nuclear/cytoplasmic fluorescence distribution are presented on the right of the image. **f** *Neat1* participates in the process of Runx2 degradation by the proteasome pathway. The WT and *Neat1*-KO osteoblasts were isolated and treated with 5 μmol·L^**−**1^ MG132 or DMSO for 6 h. The experiment was repeated three times. **g** The WT and *Neat1*-KO osteoblast lysates were immunoprecipitated with anti-Runx2 antibody. Mouse primary osteoblasts were treated with 5 μmol·L^**−**1^ MG132 for 6 h. Ubiquitination levels were analyzed by western blotting. Representative results of three independent experiments are shown. **h** Western blotting showed increased Smurf1 protein levels in the *Neat1*-KO osteoblasts, and knockdown of Smurf1 blocked the downregulation of Runx2 expression induced by *Neat1* deficiency. Representative results of three independent experiments are shown. *P < 0.05, **P < 0.01, ***P < 0.001

To elucidate the underlying mechanism of *Neat1* and paraspeckles in regulating osteoblast function, we performed RNA immunoprecipitation (RIP) assays of osteoblasts using a NONO antibody following RNA high-throughput sequencing. According to the KEGG enrichment analysis, several important pathways, including ubiquitin-mediated proteolysis, RNA transport, and protein processing, were related to the function of paraspeckles in osteoblasts, and ubiquitin-mediated proteolysis was ranked first (Fig. 4a). Subsequently, we confirmed the enrichment of candidate E3 ubiquitin ligases associated with osteoblast function by NONO RIP. q-PCR analysis showed that the indicated E3 ubiquitin ligases were sequestered in paraspeckles (Fig. 4b, Fig. S5a). Paraspeckles enable nuclear retention of mRNAs and prevent their efficient transport to the cytoplasm for translation. Therefore, we determined the changes in the protein levels of these E3 ubiquitin ligase candidates in osteoblasts with or without *Neat1* knockout. Immunoblotting showed that the Smurf1 protein level was strongly increased in the *Neat1*-KO osteoblasts (Fig. 4c). In contrast, the protein levels of other E3 ubiquitin ligases displayed no significant changes in the *Neat1-*KO osteoblasts. Moreover, analysis of nuclear/cytoplasmic RNA fractionation showed that *Smurf1* mRNA exhibited a reduced nuclear-cytoplasmic ratio in the *Neat1*-KO osteoblasts, while the total *Smurf1* mRNA remained unchanged (Fig. 4d). Subsequently, we performed RNA FISH staining and observed the colocalization of *Smurf1* mRNA and NONO in paraspeckles. However, in the *Neat1*-KO osteoblasts following paraspeckle disassembly, *Smurf1* mRNA was mainly localized in the cytoplasm (Fig. 4e). Furthermore, *Smurf1* mRNA retention in paraspeckles was identified by RNA pulldown assays with biotin-labeled DNA probes (Fig. S5b). Taken together, these data indicated that paraspeckles sequester *Smurf1* mRNA in the osteoblast nucleus.

A previous study demonstrated that Smurf1 inhibits osteoblast differentiation by targeting Runx2 for degradation.^29^ In the *Neat1*-KO osteoblasts, the Runx2 protein level was strongly decreased (Fig. 4f). Furthermore, treatment with the proteasome inhibitor MG132 restored the Runx2 protein level in the *Neat1*-KO osteoblasts to that in the WT osteoblasts. Ubiquitination assays revealed that the level of polyubiquitinated Runx2 was considerably enhanced in both the *Neat1*-KO mouse primary osteoblasts and the *Neat1* knockdown preosteoblast cell line MC3T3-E1 (Fig. 4g, Fig. S5c). To further identify the effect of Smurf1 in mediating *Neat1*-KO-induced downregulation of Runx2 expression, we disturbed Smurf1 expression with siRNA. Immunoblotting showed that Smurf1 knockdown in osteoblasts blocked the downregulation of Runx2 expression caused by *Neat1* deficiency (Fig. 4h). Thus, *Neat1* and paraspeckles regulate osteoblast function through paraspeckle retention of *Smurf1* mRNA in the nucleus, thereby reducing the Smurf1 protein level in the cytoplasm, which is involved in the regulation of protein stability of the osteoblast transcription factor Runx2.

### *Neat1* is required for the osteoblast response to mechanical stimuli

**Fig. 5:**
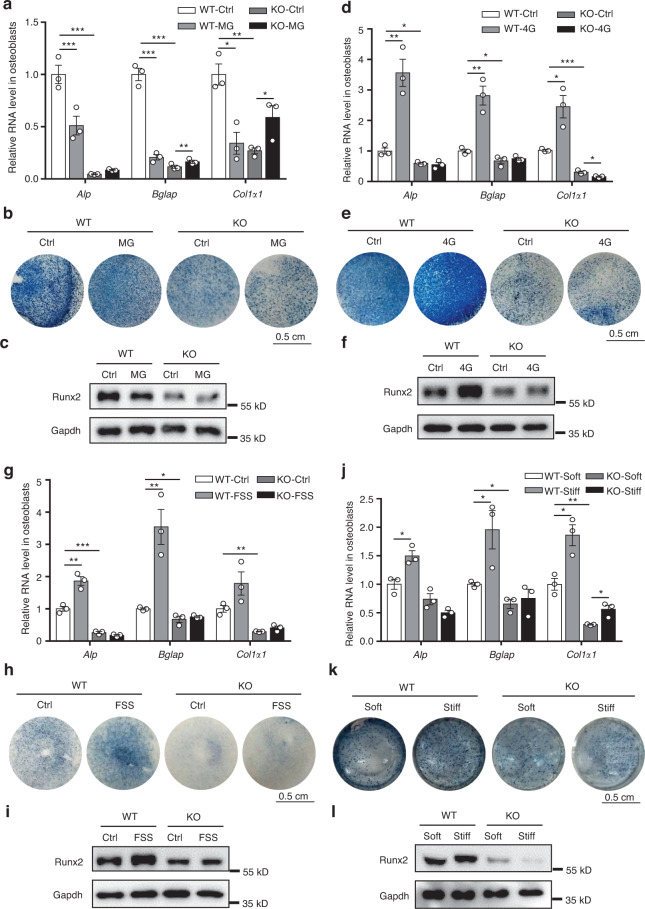
*Neat1* depletion in osteoblasts blunts their response to mechanical stimuli. **a** Analysis of the expression of the osteogenic marker genes *Alp*, *Bglap*, and *Col1α1* in the WT and *Neat1*-KO osteoblasts induced with osteogenic medium under MG conditions. The experiment was repeated for more than three times. **b** Representative results showing ALP staining of the WT and *Neat1*-KO osteoblasts induced with osteogenic medium under MG conditions. **c** Immunoblot analysis of Runx2 protein levels between the WT and *Neat1*-KO osteoblasts induced with osteogenic medium under MG conditions. **d** Expression of the osteogenic marker genes *Alp*, *Bglap*, and *Col1α1* in the WT and *Neat1*-KO osteoblasts induced with osteogenic medium under 4G conditions. The experiment was repeated more than three times. **e** Representative results showing ALP staining of the WT and *Neat1*-KO osteoblasts induced with osteogenic medium under 4G conditions. **f** Immunoblot analysis of Runx2 protein levels between the WT and *Neat1*-KO osteoblasts induced with osteogenic medium under 4G conditions. **g**, **h**. Analysis of osteogenic marker genes and ALP activity in the WT and *Neat1*-KO osteoblasts treated with FSS. The results show representative data from three independent experiments. **i** Immunoblot analysis of Runx2 protein levels in the WT and *Neat1*-KO osteoblasts after FSS treatment for 4 h. **j**, **k** Analysis of osteogenic marker genes and ALP activity in the WT and *Neat1*-KO osteoblasts cultured on soft or stiff matrix. The results show representative data from three independent experiments. **l** Immunoblot analysis of Runx2 protein levels in the WT and *Neat1*-KO primary osteoblasts cultured on soft or stiff matrix. *P < 0.05, **P < 0.01, ***P < 0.001

To confirm the essential role of *Neat1* in the osteoblast response to mechanical stimuli, we analyzed the changes in osteoblast function in the WT and *Neat1*-KO osteoblasts under different mechanical stimuli. q-PCR analysis showed that the levels of osteogenic marker genes were significantly downregulated and ALP activity was decreased in the WT osteoblasts subjected to MG treatment, whereas the *Neat1*-KO osteoblasts presented no response to MG stimuli (Fig. 5a, b). Subsequently, we determined Runx2 expression in the WT and *Neat1*-KO osteoblasts under MG conditions. Consistent with the reduced osteoblast function, Runx2 expression was strongly decreased in the WT osteoblasts treated with MG but not in the *Neat1*-KO osteoblasts compared with control osteoblasts (Fig. 5c). Moreover, under 4G hypergravity conditions, primary osteoblasts from the WT mice exhibited significantly increased expression of osteogenic marker genes (Fig. 5d), enhanced ALP activity, and upregulated Runx2 protein levels (Fig. 5e, f). In contrast, these effects were abolished in the *Neat1*-KO osteoblasts. Similarly, in the WT osteoblasts subjected to FSS or stiff matrix stimulation, osteoblast function was enhanced, which was indicated by upregulation of osteogenic marker gene expression and increased ALP activity and Runx2 expression. In contrast, regardless of the mechanical stimuli, the osteogenic markers remained unchanged in the *Neat1*-KO osteoblasts (Fig. 5g–l). These results indicated that *Neat1* in osteoblasts plays a crucial role in their responses to mechanical stimulation.

### *Neat1* KO inhibits bone formation promoted by mechanical loading

**Fig. 6:**
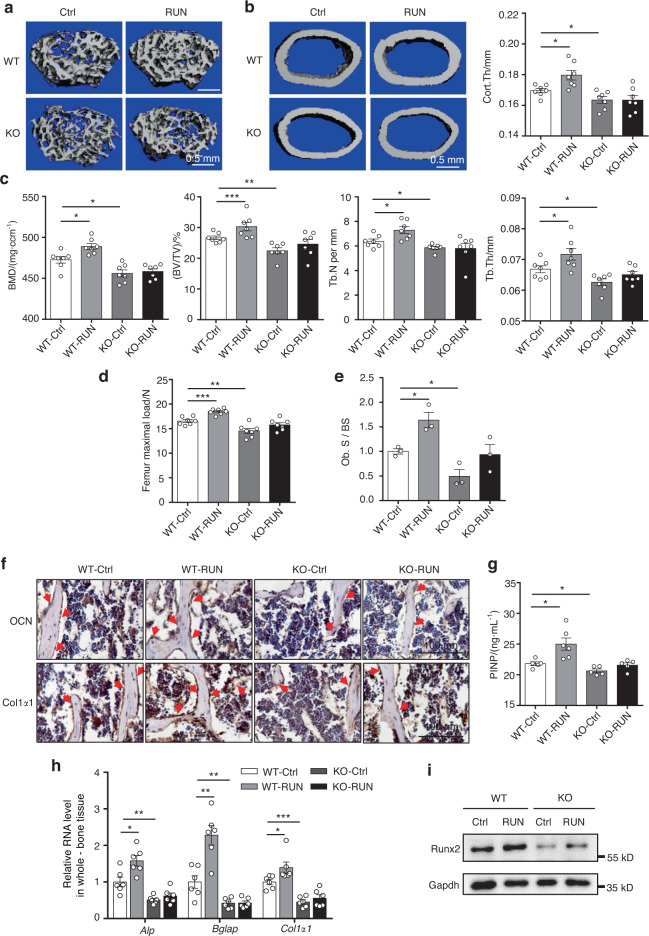
The effect of mechanical loading on the bone was significantly weakened in the *Neat1* knockout mice. **a**, **b** Representative 3D μ-CT images showing trabecular and cortical bones from distal femurs of the indicated mice. Scale bars, 0.5 mm. Cortical bone thickness of distal femurs from the control and mechanically loaded mice was analyzed. *n* = 7 in each group. **c** Quantitative µ-CT analysis of BMD, BV/TV, Tb.N and Tb.Th from 17-week-old WT and *Neat1*-KO mice. *n* = 7 in each group. **d** Femur maximal load determined by three-point bending of 17-week-old WT and *Neat1*-KO mice of the indicated groups. *n* = 7 in each group. **e** Histomorphometric analysis of osteoblast surface per bone surface in the WT and *Neat1*-KO mice with or without mechanical loading. *n* = 3 in each group. **f** Immunohistochemical staining of OCN and Col1α1 in tibial sections from 17-week-old *Neat1*-KO and control mice with or without mechanical loading. Scale bars, 100 μm. **g** ELISAs of serum PINP (ng·mL^**−**1^) in the control and *Neat1*-KO mice with or without mechanical loading. *n* = 5 or 6 in each group. **h** Osteogenic gene expression in whole-bone tissue from the WT and *Neat1*-KO mice with or without mechanical loading. *n* = 6 in each group. **i** Immunoblot analysis of Runx2 protein levels in whole-bone tissue from the WT and *Neat1*-KO mice with or without mechanical loading. Representative results of three independent experiments are shown. *P < 0.05, **P < 0.01, ***P < 0.001

Mechanical loading plays an essential role in bone formation. To further determine whether *Neat1* and paraspeckles mediate mechanical loading-induced bone formation in vivo, we subjected the *Neat1*-KO mice and their WT littermates to mechanical loading with a treadmill for 6 weeks. Micro-CT analysis of the femurs showed increased bone mass and thickness of cortical bone in the WT mice after running for 6 weeks, whereas these parameters in the *Neat1*-KO mice remained unchanged after running (Fig. 6a–c). The trabecular architecture was clearly improved in the WT mice after running, as indicated by increased BMD, BV/TV, Tb.N, and Tb.Th values. These phenotypes were inhibited in the *Neat1*-KO mice. In addition, running enhanced the maximal femur load in the WT mice. In contrast, this effect was blocked in the *Neat1* knockout mice (Fig. 6d). Furthermore, histomorphometric analysis revealed a significant increase in the osteoblast surface in the WT mice after running but not in the *Neat1*-KO mice (Fig. 6e). Consistent with this finding, the increase in OCN and Col1α1 levels in bone tissue and the PINP serum level after running were only found in the WT mice (Fig. 6f, g, Fig. S6). Furthermore, we determined the expression of osteogenic marker genes and the Runx2 protein level in the bone tissue from the running mice. As expected, mechanical loading-induced significant upregulation of osteogenic marker gene expression in the WT mice but not in the *Neat1*-KO mice (Fig. 6h). Accordingly, the changes in Runx2 showed the same trend (Fig. 6i). Taken together, these in vivo results demonstrated that *Neat1* plays an essential role in the skeletal response to mechanical loading.

### *Neat1* depletion in mice blunts mechanical unloading-induced bone loss

**Fig. 7:**
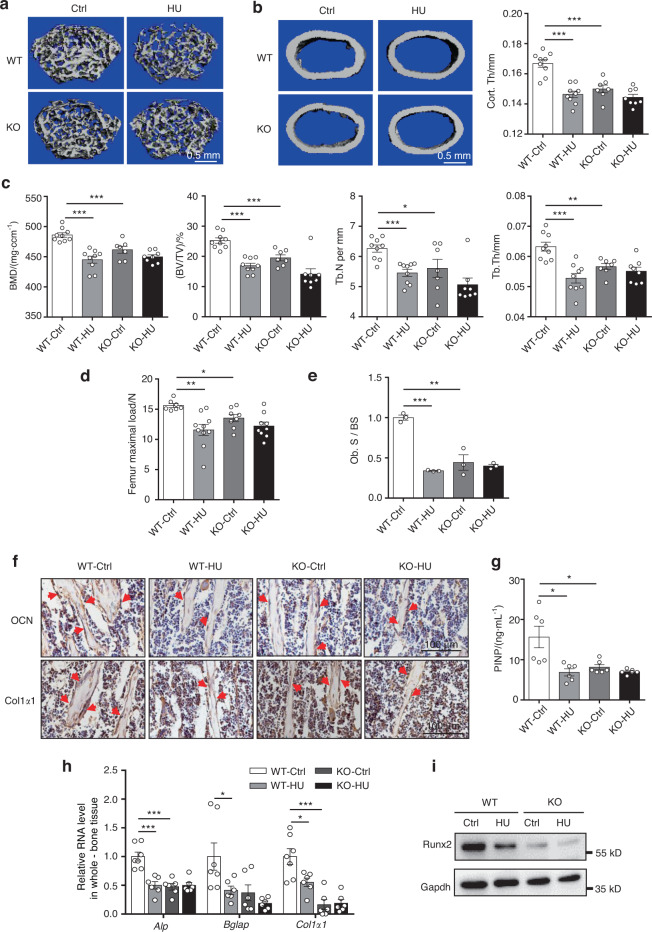
Unloading-induced bone loss was blunted in the *Neat1* knockout mice. **a**, **b** Representative 3D μ-CT images showing trabecular and cortical bones from distal femurs isolated from 15-week-old WT and *Neat1*-KO mice of the indicated groups. Scale bars, 0.5 mm. Cortical bone thickness of distal femurs from the control and HU mice was analyzed. *n* = 7–9 in each group. **c** Quantitative µ-CT analysis of BMD, BV/TV, Tb.N and Tb.Th from 15-week-old WT and *Neat1*-KO mice in the indicated groups. *n* = 7–9 in each group. **d** Femur maximal load determined by three-point bending of 15-week-old WT and *Neat1*-KO mice of the indicated groups. *n* = 7–10 in each group. **e** Histomorphometric analysis of osteoblast surface per bone surface in the WT and *Neat1*-KO mice with or without hindlimb unloading. *n* = 3 in each group. **f** Immunohistochemical staining of OCN and Col1α1 in tibial sections from 15-week-old *Neat1*-KO mice and WT mice with or without hindlimb unloading. Scale bars, 100 μm. **g** ELISAs of serum PINP (ng·mL^−1^) in the control and *Neat1*-KO mice with or without hindlimb unloading. *n* = 6 in each group. **h** The expression of osteogenic genes in whole-bone tissue from the WT and *Neat1*-KO mice with or without hindlimb unloading. *n* = 6 or 7 in each group. **i** Immunoblot analysis of Runx2 protein levels in whole-bone tissue from the WT and *Neat1*-KO mice with or without hindlimb unloading. Representative results of three independent experiments are shown. *P < 0.05, **P < 0.01, ***P < 0.001

Hindlimb unloading in mice is a typical model that is used to simulate weightlessness-induced bone loss. We subsequently examined whether *Neat1* was involved in this process, with hindlimb unloading performed in the *Neat1*-KO mice. After four weeks, the WT mice exhibited reduced tibial bone mass and impaired structures in the distal hindlimb femurs (Fig. 7a–c), as well as diminished cortical bone thickness and strength (Fig. 7b, d). Strikingly, unloading-induced changes in bone mass and structures were not found in the *Neat1*-KO mice. In the WT mice, the inhibition of bone formation induced by hindlimb unloading was also indicated by histomorphometric analysis, but this was not found in the *Neat1*-KO mice (Fig. 7e). Immunohistochemical staining for OCN and Col1α1 showed that unloading induced a reduction in osteoblast function in the WT mice but not in the *Neat1*-KO mice (Fig. 7f, Fig. S7). Serum PINP levels also revealed that the *Neat1* KO inhibited the response of osteoblasts to unloading stimulation (Fig. 7g). Consistent with this finding, the reduced expression of the osteogenic marker genes *Alp, Bglap*, and *Col1α1* and the Runx2 protein level in bone tissue indicated decreased bone formation in the WT mice subjected to hindlimb unloading but not in the *Neat1*-KO mice (Fig. 7h, i). These results collectively suggested that *Neat1* functions as a key mechanotransducer mediating unloading-induced bone loss.

## Discussion

**Fig. 8:**
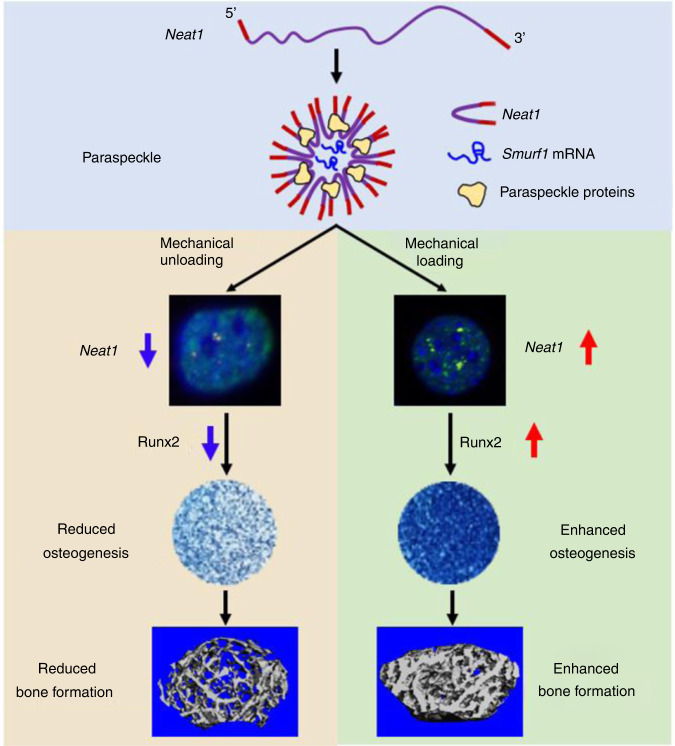
Proposed model for the regulation of *Neat1*-containing paraspeckles on osteoblasts under mechanical stimulation. *Neat1*/paraspeckle functions as an important mechanotransducer in response to mechanical stimuli and regulates bone formation (see text for details)

In this study, we demonstrated that *Neat1* acts as a novel regulator of osteoblast function and bone formation in response to mechanical stimulation through paraspeckle-dependent E3 ubiquitin ligase *Smurf1* mRNA nuclear retention. *Neat1* expression and the number and morphology of paraspeckles formed in osteoblasts changed in response to different mechanical treatments. *Neat1* deficiency following disruption of paraspeckles led to decreased osteoblast function and reduced bone mass. Depletion of *Neat1* in mice reduced the changes in bone formation in response to mechanical loading and unloading. The *Neat1*-KO osteoblasts were insensitive to different mechanical stimulations. Mechanistically, *Smurf1* mRNA was sequestered by paraspeckles in the nucleus, which prevented its translocation and translation in the cytoplasm. Accordingly, Runx2 ubiquitination and degradation were inhibited. Thus, we demonstrated a novel model in which lncRNAs act as important mediators of mechanical signal transduction during bone formation and remodeling (Fig. 8).

Mechanical stimuli exerted on the cells via the microenvironment (e.g., fluid shear stress and stiffness) or external forces (e.g., hypergravity and simulated MG) have an important impact on cell function and behavior.^30–35^ Increasing evidence has demonstrated that the nucleus acts as a mechanosensitive organelle that can sense, perceive and respond to changes in mechanical signals.^36,37^ However, the molecular mechanism remains largely unknown. Here, we screened mechanistically responsive lncRNAs and identified *Neat1* as the most significantly changed lncRNA in osteoblasts under simulated MG, which is regularly arranged with its binding protein and forms paraspeckles. We further observed that the number and morphology of paraspeckles changed with different mechanical stimulations in osteoblasts, which resulted from both *Neat1* levels and alterations in mechanical cues. In this context, we hypothesized that the mechanical forces transmitted to the nucleus through the cytoskeleton subsequently induced changes in *Neat1* expression and paraspeckles. Another possibility is that upstream ion channels mediate mechanical transduction to induce changes in intracellular chemical signals, which further affect Neat1 expression and paraspeckle formation. A further possibility is that paraspeckles directly sense the mechanical forces transmitted by the nucleoskeleton and subsequently regulate the downstream effects.

Paraspeckles enable nuclear retention of certain mRNAs with inverted repeated Alu elements or SINEs in the 3′-UTR, thereby inhibiting their translation. In addition, other unknown mRNAs are sequestered by paraspeckles. Smurf1 is a crucial E3 ubiquitin ligase for bone homeostasis, and its high expression inhibits bone development.^38^ Here, by NONO RIP and RNA pulldown experiments, we identified *Smurf1* mRNA sequestration in paraspeckles, which protects Runx2 from degradation and promotes osteoblast function.^29,39,40^ Mechanical loading-induced upregulation of *Neat1_2* expression and the assembly, condensation, and elongation of paraspeckles, leading to the upregulation of Runx2 expression and osteoblast function. In contrast, mechanical unloading reduced osteoblast function via *Neat1* suppression. The *Neat1*-KO osteoblasts lost their response to mechanical stimulation. Runx2 is the master regulator of bone formation and function,^41,42^ and bone modeling and remodeling are largely regulated by mechanical cues.^5,6^ Our findings established the important role of *Neat1* in the association of mechanical signals with osteoblast regulation and demonstrated how lncRNA *Neat1* performs its unique regulation of osteoblasts in response to different mechanical stimulations. Dynamic assembly and disassembly of paraspeckles and the precise regulation of mRNA translocation make osteoblasts highly flexible in response to different stimuli. However, the mechanism of *Smurf1* mRNA retention in paraspeckles requires further investigation.

LncRNAs play key roles in the regulation of biological and physiological processes, including osteogenesis,^43^ bone resorption,^44^ skeletal aging,^45^ and osteoporosis.^44^ In this study, we revealed the specific role of lncRNA *Neat1* in bone formation and the occurrence of osteoporosis. *Neat1* depletion resulted in disrupted bone formation, severe bone loss, and reduced responses to mechanical loading. *Smurf1* retention in the paraspeckles mediated the important role of *Neat1* in loading-induced bone remodeling. Bone loss induced by unloading is a serious problem normally caused by long-term spaceflight or prolonged bed rest. The rate of bone loss in MG is approximately ten times greater than the bone mineral density loss per month that occurs in postmenopausal women. In spaceflight, the changes in bone structure are resistant to complete recovery and mimic the changes in the elderly. However, the currently available pharmaceutical countermeasures and exercise cannot completely prevent bone loss. It is important to test new therapies to address this issue. Increasing evidence has suggested that noncoding RNAs may be drug targets. In our study, *Neat1* was identified as the most mechanosensitive lncRNA. Strikingly, the unique structure of the paraspeckle in the nucleus makes it more suitable to perform roles that cannot be achieved by DNA or protein, and it acts as a spatial amplifier of regulatory signals in the nucleus.

Our results demonstrated that paraspeckle functions as a key mechanotransducer structure in the nucleus to confer mechanosensitivity to osteoblasts. Here, treadmill running is an exercise model that enhances bone strength and mass. A more direct mechanical loading device can also be used on the bone to investigate the role of paraspeckle in osteoblast function. Unloading-induced bone loss or osteoporosis may be eliminated by upregulating the *Neat1* level or stabilizing the paraspeckle structures through exercise and mechanical loading in combination with specific small molecules and oligonucleotides. Taken together, our findings not only provide a novel working model for lncRNAs in response to mechanical signals but also supply a therapeutic target for treating osteoporosis and unloading-induced bone loss.

## Methods

### Cell culture

Primary osteoblasts were isolated from the calvaria of neonatal mice within 4 days. In brief, mouse calvaria were dissected aseptically and digested with 0.2% collagenase in minimum essential medium alpha (α-MEM) for 30 min. The digested cells were seeded in a culture flask with α-MEM containing 10% FBS (Gibco, USA) and 1% penicillin and streptomycin (Gibco, USA). The MC3T3-E1 cell line was obtained from NICLR (Beijing, China) and cultured according to the recommendations of ATCC. Ascorbic acid (50 μg·mL^**−**1^, A4403, Sigma) and 5 mmol·L^**−**1^ β-glycerophosphate (G9422, Sigma) were used for primary osteoblast osteoblastic differentiation.

### RNA-seq and analysis

For mRNA-seq, raw data in FASTQ format were analyzed using fastp (version 0.19.11) software. BWA (v 0.7.12) was used to build an index of the reference genome and align clean reads to the reference genome. Regions of IP enrichment over background were identified with MACS2 (version 2.1.0) peak calling software. Peak-related genes were confirmed by PeakAnnotator, and we performed KEGG (Kyoto Encyclopedia of Genes and Genomes, http://www.genome.jp/kegg/) enrichment analysis to identify the pathway map results. Then, the statistical enrichment of peak-related genes in KEGG pathways was tested with KOBAS software.

For lncRNA-seq, an RNA library was prepared using the rRNA depletion and stranded method, and 12 G raw data were obtained using PE150 (paired-end 150 nt) sequencing. FASTQ format raw data were first processed through in-house Perl scripts for clean reads that were mapped to a reference genome with HISAT2 software, and then, transcripts were assembled with StringTie. LncRNAs were identified after all the transcripts were merged with Cuffmerge software. StringTie software was used to quantify the transcripts and genes to reads per kilobase of transcript per million mapped reads. Finally, Cuffdiff or edgeR was used for differential expression analysis. The false discovery rate was controlled according to Benjamin Hochberg adjusted *P* values. Differentially expressed genes were defined as those with a standard *P* < 0.05 and fold change > 1.3.

### Rotation-simulated microgravity

A two-dimensional (2D) clinostat was used to simulate the effects of MG, as described in our previous studies.^35,46^ Briefly, osteoblasts were incubated in T-12.5 culture flasks or plated on 25 mm glass coverslips for RNA extraction or FISH image acquisition. The parameters set on clinostat were 0.01 g (equivalent to the MG of low earth orbit) at 37 °C for 48 h. Osteoblasts without rotation were cultured in the same manner as the control group.

### Hypergravity centrifuge

A hypergravity centrifuge was used to detect the effects of hypergravity on osteoblasts, as described in our previous study.^46^ The detailed procedure of cell preparation was the same as rotation-simulated MG. The parameters of the hypergravity centrifuge were set as 4G at 37 °C for 48 h.

### Fluid shear stress experiment

Fluid shear stress was generated as described in our previous study.^35^ Briefly, osteoblasts were seeded on 22 × 26 mm glass coverslips. The coverslips were placed into holders when the cell density reached 80%. Fluid flow stress was applied to the cells by the medium flowing past glass coverslips in the parallel plate flow chamber in a closed flow loop at 37 °C for 4 h.

### Preparation of soft and stiff matrix

Two-dimensional culture on matrix gel of high (40.40 ± 2.39 kPa) or low (1.00 ± 0.31 kPa) stiffness was performed according to the manufacturer’s directions for advanced BioMatrix (GS1005) and the description in the ref. ^47^ In brief, soft and stiff hydrogel solutions consisted of 1:5 ratios of 5 mg·mL^**−**1^ Extralink: (10 mg·mL^**−**1^ Glycosil + 10 mg·mL^**−**1^ Gelin-S) or 25 mg·mL^**−**1^ Extralink: (20 mg·mL^**−**1^ Glycosil + 20 mg·mL^**−**1^ Gelin-S). The mixture was then added to glass coverslips or 48-well plates for cell culture.

### Mice

The generation strategy of *Neat1*-KO mice (provided by Mian Wu, University of Science and Technology of China, China) was described in detail in ref. ^47^ All mice were maintained and bred in a specific pathogen-free facility, and animal experiments were undertaken and conducted in accordance with institutional guidelines and approval from the Committees of Animal Ethics and Experimental Safety.

### Human bone tissue

Normal bone tissue and osteoporotic bone tissue samples were both collected in clinical settings from patients older than 60 years who underwent surgery at the Second Affiliated Hospital of Soochow University. According to the National Osteoporosis Foundation, osteoporosis is defined as a bone mineral density (BMD) T score of ≤−2.5, and a T score of ≥−2 is considered normal. The inclusion criteria were previously described.^48^ Patients who were subjected to diabetes, malignancy, hyperparathyroidism, and other severe bone diseases were excluded from our study. We also excluded patients who had taken glucocorticoids, estrogen, or anti-osteoporosis drugs within 1 year. Dual-energy X-ray absorptiometry was used for BMD detection in the hip and spine. We obtained all participants’ informed consent. The study protocol conformed to the ethical guidelines of the 1975 Declaration of Helsinki and was approved by the Committees of Clinical Ethics of the Second Affiliated Hospital of Soochow University (Reference number: 2016 K-22).

### Mouse model of hindlimb unloading

Tail suspensions were performed to mimic weightlessness-induced bone loss by removing body weight-induced mechanical loading on hind limbs.^49^ Each mouse was bred in a single cage, and hindlimb unloading was attained by hanging the tail on the chain with pulley by adhesive surgical tape until the angle between the body of the mouse and the floor reached 30°. The mouse was allowed to move and access food and water freely. After tail suspension for 28 d, the mice were euthanized, and bilateral femurs and tibiae were dissected for micro-CT examination, bone histomorphometry analysis and q-PCR analysis.

### Exercise mouse model

Eleven-week-old WT and *Neat1*-KO male mice were randomly divided into four groups for control and exercising. The exercising group was placed on a treadmill with a 10° incline at 13 m·s^**−**1^ for 30 min. After the mice repeatedly exercised 5 days a week for 6 weeks, bilateral femurs and tibiae were dissected for bone histomorphometry analysis and gene expression analysis at the end of the experiment.

### Immunohistochemistry and bone histomorphometry

After fixation with 4% PFA for 48 h, the tibias from the WT and *Neat1*-KO mice were maintained in 18% EDTA solutions for 4 weeks for decalcification and then embedded in paraffin. According to the IHC protocol, paraffin-embedded slices were dewaxed in xylene and rehydrated in graded alcohol solutions. Then, antibodies against osteocalcin (1:500, Proteintech, 23418-1-AP) or Col1α (1:200, Abcam, ab96723) were used for staining. Quantitative histomorphometric analysis was performed on the tibias of male mice, and osteoblast number per bone surface was measured by conducting toluidine blue staining using OsteoMeasure XP Software (OsteoMetric) in a blinded fashion.

### Micro-CT analysis

The bone phenotype of the WT and *Neat1*-KO mice was analyzed on the femur by a micro-CT system (Scanco Medical, μ-40, Switzerland). Briefly, for each distal femur, 634 slices with a voxel size of 10.5 µm were scanned, and eighty continuous interest slices were selected for analyzing the trabecular bone at a distance of 210 μm from the growth plate. For cortical measurements, eighty continuous slices of interest in the diaphyseal region starting at a distance of 3.57 mm from the growth plate were selected. The parameters BMD, BV/TV, Tb.N, Tb.Th, Tb.Sp and Cort.Th were calculated from the three-dimensional reconstruction of selected slices.

### Double calcein labeling

Peritoneal injection with calcein (30 mg·kg^−^^1^ body weight) was performed 10 and 2 days before mouse euthanasia. Tibias with soft tissues removed were harvested and fixed in 4% PFA. Images were collected with confocal microscope. Data were analyzed using OsteoMeasurexp^TM^ (version 1.01) software.

### Whole-mount staining

Skeletal preparations were stained with ARS and Alcian blue as previously described.^50^ In brief, newborns were collected, and the skin was removed. The samples were fixed with 95% ethanol for 72 h and incubated with acetone for 48 h. Subsequently, the samples were stained in Alcian blue solution for 3 days. Then, they were cleared with 75% ethanol three times for 1.5 h each, followed by treatment with 1% KOH overnight. After staining with 0.005% ARS solution for 5 h, the samples were cleared and stored in 1% KOH/20% glycerol.

### Three-point bending analysis

For the three-point bending test, the femurs were dissected, and soft tissues were removed. The measurement was performed at the middle of the femur by the experimental setup of the Texture Analyzer (serial number: 8540307). Texture Pro CT software was used for further analysis.

### Measurement of serum PINP concentrations

We measured serum concentrations of PINP using a PINP (procollagen type I N-terminal propeptide) ELISA kit (ImmunoWay, KE1744) according to the manufacturer’s instructions.

### Alkaline phosphatase staining and Alizarin red staining

Primary osteoblasts were fixed using 4% PFA and washed with PBS three times at room temperature. ALP staining was performed with a Vector Blue Substrate Kit (Vector Laboratories, SK-5300). Osteoblasts were incubated with working solution for 1 h and then rinsed with PBS. For Alizarin red staining, osteoblasts were stained with 40 mmol·L^**−**1^ Alizarin red S (ARS, Sigma-Aldrich, A-5533) at pH 4.0. Then, osteoblasts were rinsed with double-distilled H_2_O five times. Both of the staining procedures were performed protected from light.

### Transient transfection

Plasmid and siRNA transfection were carried out with Lipofectamine 3000 (Invitrogen, L3000-015) or RNAi Max (Invitrogen, 13778-150) according to the manufacturers’ protocol. The siRNA sequence used in the study are listed: Mouse negative control (NC) siRNA: 5′-UUCUCCGAACGUGUCACGUTT-3′; Mouse *Neat1* siRNA: 5′-GGAGUCAUGCCUUAUACAATT-3′; Mouse *Neat1_2* siRNA: 5′-GAGTTACCATCCCGTCCTCTATT-3′; Mouse *NONO* siRNA: 5′-TTCCCTGCTTGTACTACTCTA-3′; Mouse *PSF* siRNA: 5′-CAGUCAUUGUGGAACCACUUGAACA-3′; Mouse *Smurf1* siRNA:5′-UGAAGAAGUCUUUCUUUGCAA-3′.

### IP and western blotting

Immunoprecipitation was performed as previously described.^48^ MC3T3-E1 cells or primary osteoblasts were treated with 5 μmol·L^**−**1^ MG132 (Sigma) for 6 h. Cells were lysed in buffer (150 mmol·L^**−**1^ NaCl, 50 mmol·L^**−**1^ Tris, pH 7.8, 10% glycerol, 1 mmol·L^**−**1^ EGTA, 0.5% NP-40, 1 mmol·L^**−**1^ EDTA, 1 mmol·L^**−**1^ PMSF, 1× cocktail) on ice with vigorous shaking for 30 min. The lysates were centrifuged at 4 °C at 12 000 r·min^**−**1^ for 15 min. Twenty microliters of lysate was saved as the total protein sample. The rest of the supernatant was incubated with 20 μL of protein A/G (Santa Cruz) for 40 min to exclude nonspecific binding. After 10 min of centrifugation at 12 000 r·min^**−**1^, the supernatant was subjected to IP with Runx2 antibody (Cell Signaling Technology, 12556S) and protein A/G at 4 °C for 4 h. The antibody and protein A/G were incubated for 2 h in advance. Finally, the protein A/G beads were collected and washed with cell lysis buffer twice. The lysates were subjected to western blotting with the indicated antibody. The following antibodies were used in the study: ubiquitin (1:2 000, Cell Signaling Technology, 3936S), GAPDH (1:5 000, Abways Technology, AB0036), Runx2 antibody (1:1 000, CST, 12556S), NONO (1:1 000, Abways Technology, CY8525), SFPQ (1:1 000, Abways Technology, CY8089), Smurf1 (1:1 000, Santa Cruz, sc-100616), and HA-tag (1:2 000, Cell Signaling Technology, #3724S).

### RNA fluorescence in situ hybridization and immunofluorescence microscopy

For preparation of probes, the T7 promoter was added to the template by PCR amplification. PCR products were purified and used as templates for in vitro transcription (ITC) using a fluorescein (FITC) or digoxigenin (DIG) RNA labeling kit (Roche). For detection of *Neat1* localization, osteoblasts on confocal dishes or coverslips were rinsed gently in PBS and then fixed in 4% fixative solution (Solarbio, P1110) for 15 min at room temperature. After three rinses with PBS, the cells were permeabilized in 0.5% Triton X-100/PBS on ice for 10 min and washed three times with PBS. Subsequently, the cells were incubated with prehybridization solution [50% formamide (Sigma, F9037), 2× SSC (Invitrogen), 1× Denhardt’s solution (Sigma-Aldrich), 10 mmol·L^**−**1^ EDTA pH 8.0, 100 μg·mL^**−**1^ yeast tRNA (Sigma), and 0.01% Tween-20] at 55 °C for 1.5 h and then incubated with hybridization solution [50% formamide, 2× SSC, 1× Denhardt’s solution, 10 mmol·L^**−**1^ EDTA, 100 μg·mL^**−**1^ yeast tRNA, 5% dextran sulfate (Sigma-Aldrich), and 0.01% Tween-20] containing DIG- or FITC-labeled RNA probes at 5–10 μg·mL^**−**1^ for 18–20 h at 55 °C. The probes were denatured at 100 °C for 10 min before incubation. After hybridization, the cells were washed three times with prewarmed wash buffer (50% formamide, 2× SSC and 0.01% Tween-20) at 55 °C for 10 min. Then, the cells were washed 3 times with buffer (2× SSC and 0.01% Tween-20) at 55 °C for 10 min and then washed 3 times with buffer (0.2× SSC and 0.01% Tween-20) at 55 °C for 10 min. Next, the cells were washed with 1× TBST (150 mmol·L^**−**1^ NaCl, 20 mmol·L^**−**1^ Tris pH 7.5, and 0.01% Tween-20) for 5 min and incubated with 1× blocking buffer [blocking reagent (Roche) and 1× TBST] for 1 h at room temperature. Next, the cells were incubated with DIG (anti-DIG antibody, Abcam, ab420), FITC (anti-FITC antibody, Abcam, ab19491), or NONO (NONO/p54nrb antibody, Abways, CY8525) antibodies in 1× blocking buffer at RT for 1 h, washed 3 times with TBST for 5 min, incubated with secondary antibodies (FITC-labeled goat anti-rabbit IgG, Origen, ZF-0311; TRIC-labeled goat anti-mouse IgG, ORIGEN, ZF-0313) in 1× blocking buffer at RT for 1 h, and washed three times with 1× TBST for 5 min. Finally, cell nuclei were stained with Hoechst (Sigma-Aldrich) and washed with PBS 3 times. Images were taken with a Zeiss LSM 710 microscope for confocal scans and Zeiss 880 Elyra S1 for SIM image acquisition. ZEN3.1 (blue edition) was used for image processing and paraspeckle area analysis.

### RNA extraction and q-PCR analysis

Osteoblast and bone tissue RNA was extracted with TRIzol reagent (Life Technologies, 15596018) according to the manufacturer’s instructions. RNA was reverse transcribed with the PrimeScript RT reagent Kit (TaKaRa, China) according to the manufacturer’s instructions. Quantitative reverse transcriptase-PCR (q-PCR) was carried out using a SYBR Premix Ex Taq II Kit (TaKaRa, China). The mRNA level was normalized to the *Gapdh* mRNA level using the 2^−ΔΔCT^ method. All primers are listed in Table S1.

### Cytosolic and nuclear fractionation

Cell pellets were collected and suspended in 250 μL of lysis buffer [140 mmol·L^**−**1^ NaCl, 10 mmol·L^**−**1^ Tris, pH 8.0, 1.5 mmol·L^**−**1^ MgCl_2_, 0.5% Igepal (Sigma-Aldrich, l3021), 2 mmol·L^**−**1^ vanadyl ribonucleoside complex (VRC, Sigma, 94740)] and incubated for 5 min on ice. Then, 20 μL of lysate was saved as total RNA. The supernatant was centrifuged for 5 min at 1 000 × *g* and collected as the cytoplasmic fraction. The supernatant was further centrifuged at 13 000  r·min^**−**1^ for 10 min at 4 °C and combined with TRIzol reagent for RNA extraction. The nuclear pellets were washed 3 times and then suspended in 150 μL of lysis buffer (150 mmol·L^**−**1^ NaCl, 50 mmol·L^**−**1^ Tris pH 7.4, 0.5% Igepal) followed by RNA extraction.

### RNA immunoprecipitation

Osteoblasts in a T-75 flask were harvested after reaching 95% confluency. The cells were washed with ice-cold PBS and suspended in 800 μL of RIP buffer (150 mmol·L^**−**1^ NaCl, 50 mmol·L^**−**1^ Tris pH 7.4, 0.5% Igepal, 2 mmol·L^**−**1^ VRC, 1 mmol·L^**−**1^ PMSF and 1× protease inhibitor cocktail) supplemented with RNase inhibitor (TaKaRa, 2313 A). After incubation on the rotating wheel for 30 min at 4 °C, the lysates were centrifuged at 4 °C at 1 000 × *g* for 10 min, and the supernatants were precleared with 20 μL of Protein G PLUS-agarose beads (sc-2002, Santa Cruz). The precleared supernatants were equally divided into two parts and incubated with 30 μL of Protein G PLUS-agarose beads with NONO antibodies (BD Biosciences, 611278) or mouse IgG2b (Abcam, ab18421) for 3 h at 4 °C, followed by washing with RIP buffer three times. Finally, the precipitates were dissolved in TRIzol reagent for RNA isolation.

### RNA pulldown

RNA pulldown was performed as described in a previous study^51^ with the probes below. Primary osteoblasts were harvested and suspended in RIP buffer. For pulldown with biotin-labeled DNA probes, primary osteoblast lysates were incubated with biotinylated antisense or sense DNA oligomers corresponding to *Neat1* for 2 h. Then, 20 μL of streptavidin-coupled agarose beads were added and incubated for 1 h. After washing with lysis buffer, the precipitated complexes were dissolved in TRIzol reagent for RNA abstraction. *Neat1-*1-sense (biotin-)TGACAAGGAGGGCTCGCTCTT *Neat1-*1*-*antisense (biotin-)AAGAGCGAGCCCTCCTTGTCA *Neat1-*2*-*sense (biotin-)GCACAAGTTTCACAGGCCTAC *Neat1-*2-antisense (biotin-)GTAGGCCTGTGAAACTTGTGC *Neat1-*3-sense (biotin-)GACACCCTGACTGGGCGGGGA *Neat1-*3-antisense (biotin-)TCCCCGCCCAGTCAGGGTGTC *Neat1-*4-sense (biotin-)CACTATAGTGTTCACCATGGC *Neat1-*4-antisense (biotin-)GCCATGGTGAACACTATAGTG *Neat1-*5-sense (biotin-)TTCTGGACTAAAAGGGATCCG *Neat1-*5-antisense (biotin-)CGGATCCCTTTTAGTCCAGAA *Neat1-*6-sense (biotin-)GGTTGTCACTACCCTGACCTA *Neat1*-6-antisense (biotin-)TAGGTCAGGGTAGTGACAACC

### Study approval

We maintained all animals in the animal facility of the China Astronaut Research and Training Center. All animal experimental procedures in the study were approved by the Committees of Animal Ethics and Experimental Safety of ACC (ACC-IACUC-2017-003).

### Statistical analysis

GraphPad Prism version 6 software (Nashville, TN, USA) was used for statistical analysis. All data were generated from three independent replicates. For animal analyses, at least five mice were assigned to each experimental group. Statistical comparisons were performed with a two-tailed unpaired *t* test or one-way analysis of variance. All quantitative data used in this study are presented as the mean ± standard error of the mean. Differences were considered significant at *P < 0.05, **P < 0.01, and ***P < 0.001.

## Supplementary information

Mechanosensitive lncRNA Neat1 promotes osteoblast function through paraspeckle-dependent Smurf1 mRNA retention

## Data Availability

All the data supporting the findings of this work are available from the corresponding author on reasonable request. RNA-seq raw data have been uploaded to the China National GeneBank (CNGB). The project accession number is CNP0002309.
