# Supplementary material for: The mechanosensitive lncRNA Neat1 promotes osteoblast function through paraspeckle-dependent Smurf1 mRNA retention
Source: Bone Res. 2022 Feb 24;10:18. doi: 10.1038/s41413-022-00191-3 (PMC8873336; doi:10.1038/s41413-022-00191-3)
Supplement: Supplementary file 1 — Mechanosensitive lncRNA Neat1 promotes osteoblast function through paraspeckle-dependent Smurf1 mRNA retention [file 41413_2022_191_MOESM1_ESM.pdf]

Supplementary Figure 1

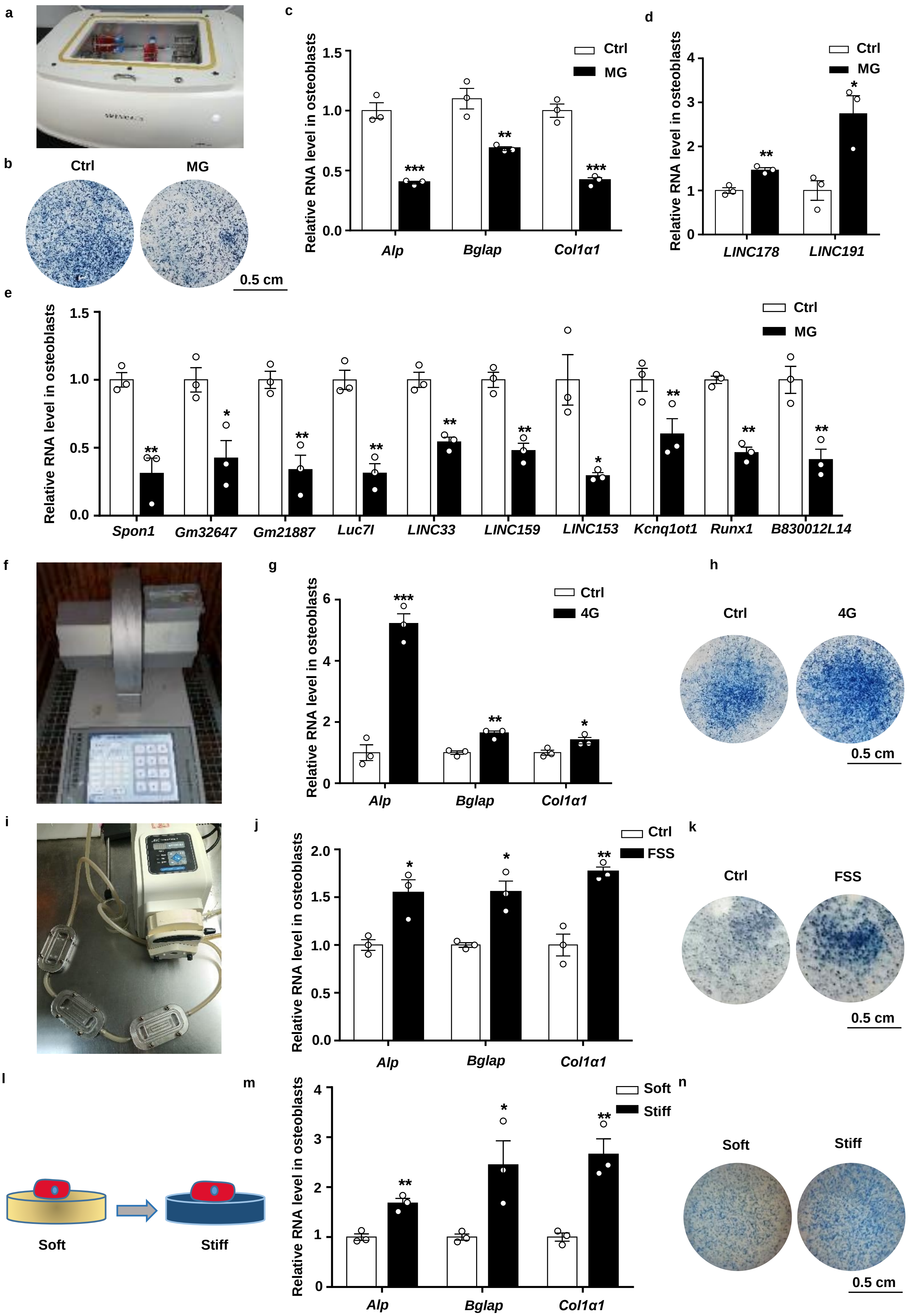

Fig. S1. Changes of osteoblasts function under different mechanical stimulation models.

a. The image of clinostat device that used to simulate microgravity. b. ALP staining of osteoblasts induced with osteogenic medium under MG condition. c. Q-PCR analysis of osteoblast marker genes *Alp*, *Bglap* and *Col1α1* expression after MG treatment. Representative results of three independent experiments are shown. d. Q-PCR validation of upregulated lncRNAs in RNA-Seq result. e. Q-PCR validation of downregulated lncRNAs in RNA-Seq result. f. The image of hypergravity centrifuge. g. Q-PCR analysis of osteoblast marker genes *Alp*, *Bglap* and *Col1α1* expression after 4G treatment. Representative results of three independent experiments are shown. h. ALP staining of osteoblasts induced with osteogenic medium under 4G condition. i. The image of fluid shear stress (FSS) system. j. Q-PCR analysis of osteoblast marker genes *Alp*, *Bglap* and *Col1α1* expression after FSS treatment. Representative results of three independent experiments are shown. k. ALP staining of osteoblasts induced with osteogenic medium under FSS condition. l. Model of soft and stiff matrix. m. Q-PCR analysis of osteoblast marker genes *Alp*, *Bglap* and *Col1α1* expression in osteoblasts cultured on soft or stiff matrix. Representative results of three independent experiments are shown. n. ALP staining of osteoblasts cultured on soft or stiff matrix with osteogenic medium. *$P < 0.05$, **$P < 0.01$, ***$P < 0.001$.

Supplementary Figure 2

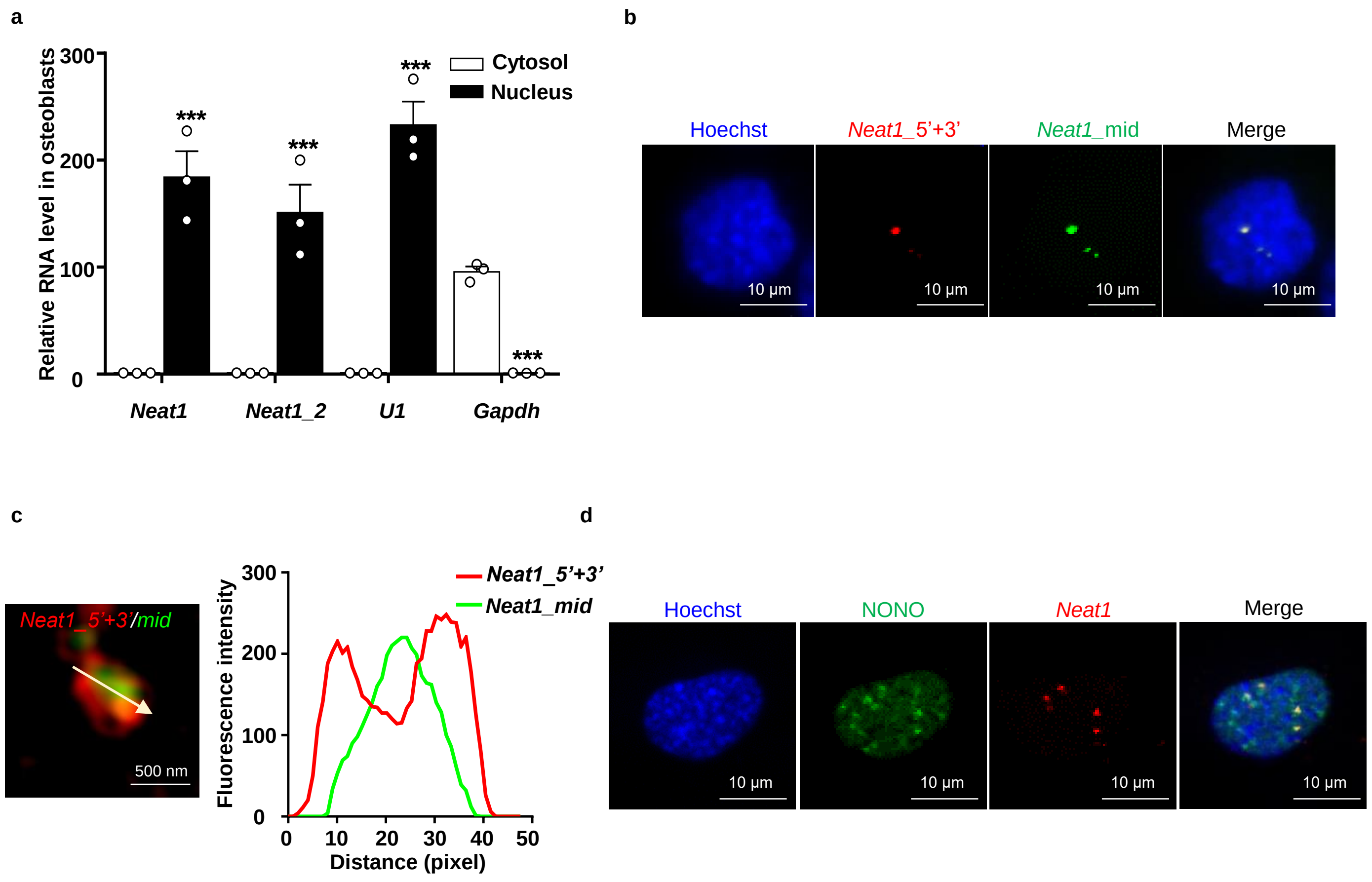

Fig. S2. The location of *Neat1* in osteoblasts.
a. Q-PCR analysis of *Neat1* and *Neat1_2* in osteoblasts by nuclear and cytoplasmic separation. *U1* and *Gapdh* were used as positive control. b. Representative images of confocal observations of paraspeckles. c. The core shell pattern of paraspeckles under SIM. d. Simultaneous detection of *Neat1* and NONO in osteoblasts by confocal microscopy. Representative results of three independent experiments are shown. ***$P < 0.001$.

Supplementary Figure 3

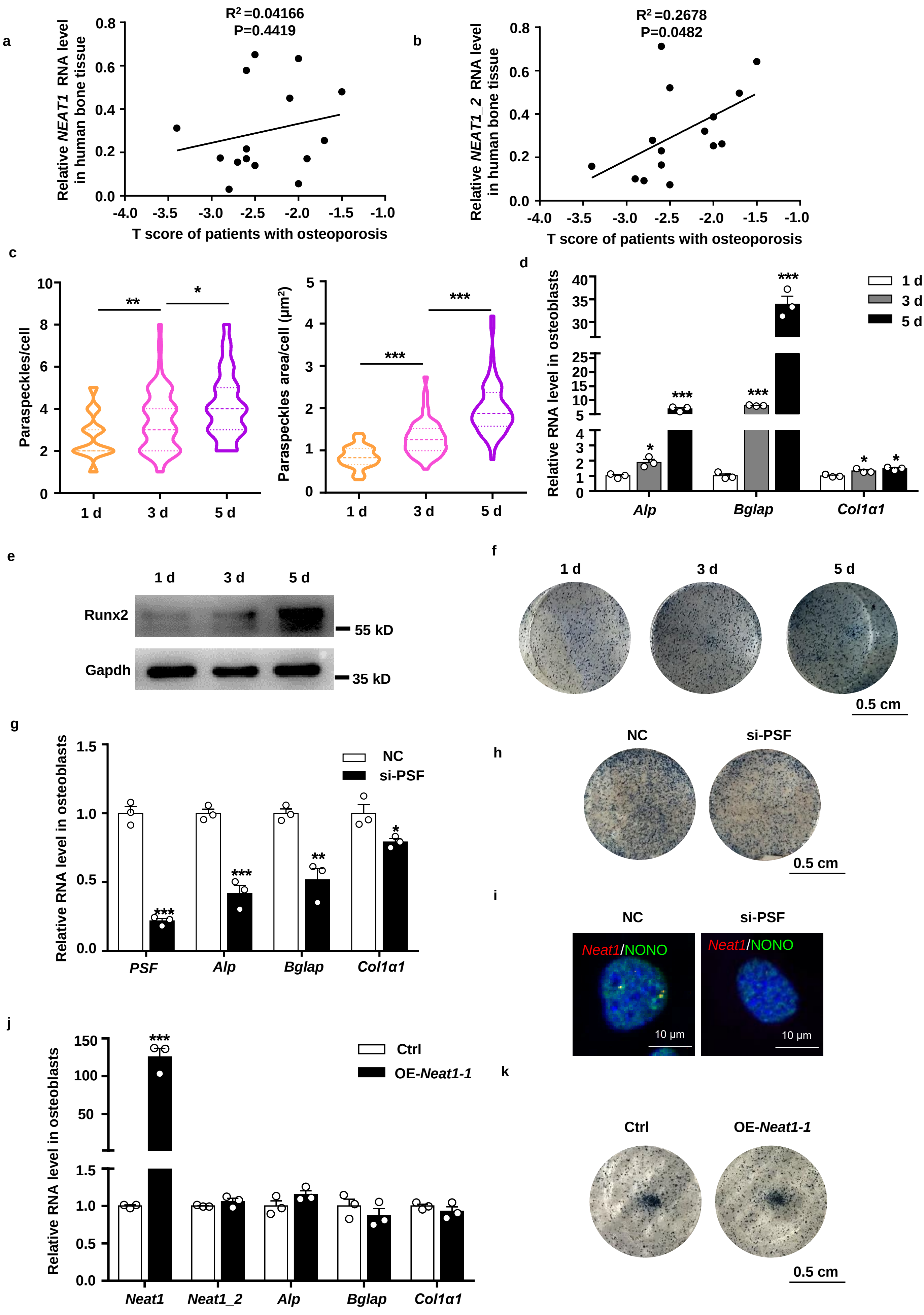

Fig. S3. The correlation of paraspeckle components and osteoblast function.

a-b. Correlation analysis between *Neat1*, *Neat1_2* and T score in bone specimens from patients with osteoporosis. c. Quantative analysis of paraspekles number and area in the process of osteoblast differentiation. d. Q-PCR analysis of osteoblast marker genes *Alp*, *Bglap* and *Col1α1* expression in the process of osteoblast differentiation. e. Western blotting showed increase expression of Runx2 during the process of osteoblast differentiation. f. Increased ALP activity was detected in the process of osteoblast differentiation. g. Q-PCR analysis of osteoblast marker genes *Alp*, *Bglap* and *Col1α1* expression after paraspeckles protein PSF knockdown. h. Representative images of ALP staining after osteoblasts transfected with NC or *PSF* siRNA. i. Representative FISH images for paraspeckles in osteoblasts upon NONO knockdown or not. j. Q-PCR analysis showed no changes of osteoblast marker genes after *Neat1_1* overexpression. k. *Neat1_1* overexpression had no effect on ALP activity by ALP staining. Representative results of three independent experiments are shown. *$P < 0.05$, **$P < 0.01$, ***$P < 0.001$.

Supplementary Figure 4

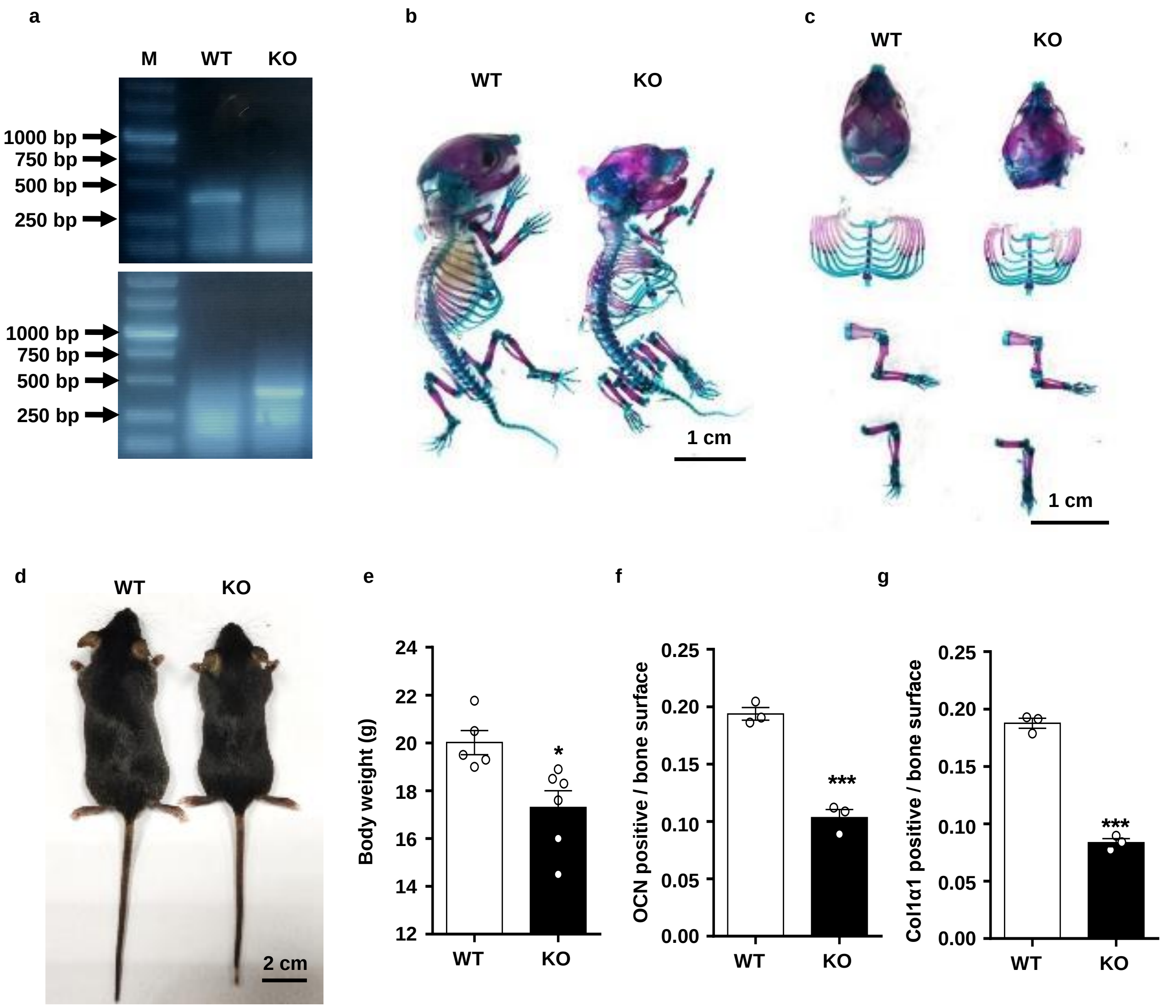

Fig. S4. The whole-mount skeleton staining, body weight and osteoblast marker proteins quantification of *Neat1* knockout mice.

a. Genotyping of WT and *Neat1*-KO mice. M, DNA marker. b-c. Whole mount skeleton staining of 2-day-old WT and *Neat1*-KO mice by Alcian blue and Alizarin red S. Scale bar = 1 cm. d. Representative image of *Neat1*-KO mice compared with the littermate. e. Body weight analysis of 5-week-old *Neat1*-KO (n=6) mice and littermate (n=5). f-g. Quantification of OCN and Col1α1 on trabecular bone surface in proximal tibia from WT and Neat1-KO mice. *$P < 0.05$, **$P < 0.01$, ***$P < 0.001$.

Supplementary Figure 5

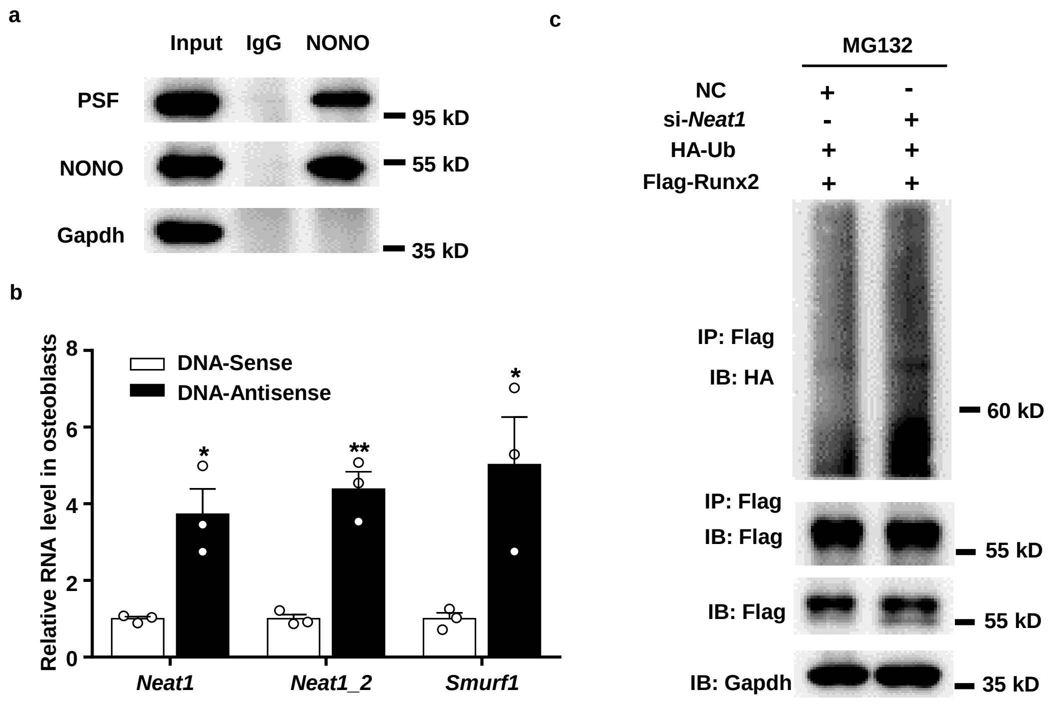

Fig. S5. The verification of ubiquitin mediated proteolysis pathway in *Neat1* deficient osteoblasts.

a. Immunoblot analysis of NONO and PSF protein enrichment in NONO-RIP samples. b. Q-PCR showed increased *Smurf1* mRNA abundance in *Neat1* antisense pulldown group compared with sense control. c. Effect of *Neat1* on the ubiquitination of exogenous Runx2. NC and *Neat1* siRNA were transfected into MC3T3-E1 when cells seeded into the plate. HA-Ub and Flag-Runx2 were transfected after 6-8 h. The cells were treated with 5 μM MG132 for 6 h, and Runx2 ubiquitination was analyzed by western blotting using an anti-HA antibody. Representative results of three independent experiments are shown.

Supplementary Figure 6

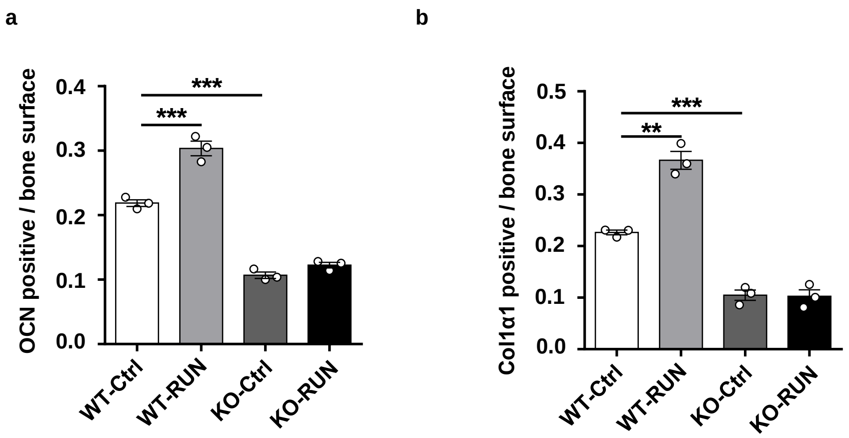

Fig. S6. Osteoblast markers quantification of WT and *Neat1* knockout mice after mechanical loading.

a-b. Quantification of OCN and Col1α1 on trabecular bone surface in proximal tibia from WT and Neat1-KO mice after mechanical loading or not. **$P < 0.01$, ***$P < 0.001$.

Supplementary Figure 7

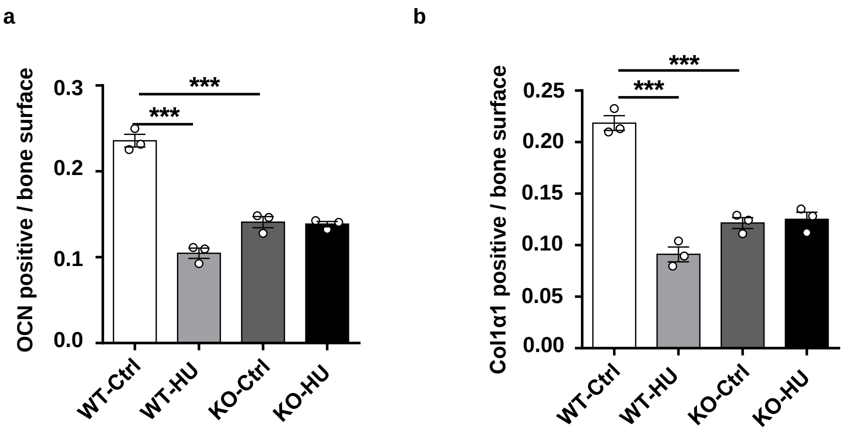

Fig. S7. Osteoblast markers quantification of WT and *Neat1* knockout mice after hind-limb unloading.

a-b. Quantification of OCN and Col1α1 on trabecular bone surface in proximal tibia from WT and Neat1-KO mice after hind-limb unloading or not. $***P < 0.001$.

Supplementary Table 1. Specific primers	
**Mouse**	
Primer	Sequence(5'to3')
Mmu-LINC178-F	CCCTAGGGCTTTCTGGCATT
Mmu-LINC178-R	GGCAGATGGATCAGTTGCCT
Mmu-LINC191-F	ATGCCCTTGGTGGCATCTTC
Mmu-LINC191-R	GGAGCGGGCATTCCTAGAG
Mmu-Spon1-F	CAGATAGGCCCACGTTCCAG
Mmu-Spon1-R	AGGAAACCGAGGATGCTTGAG
Mmu-Gm32647-F	CTGGCAAGAAGGCACCCTAT
Mmu-Gm32647-R	AATTGACCTGCAGCAAGCAC
Mmu-Gm21887-F	CACAGTGATGTCACCCACGA
Mmu-Gm21887-R	CAGGCTTCCTACCTTGTGGA
Mmu-Luc7l-F	CTCGGGACGGTTCAGTGTATC
Mmu-Luc7l-R	TTGTGACTCACGGCTCGATG
Mmu-LINC33-F	CTCACATAGCCCAGTAGCCC
Mmu-LINC33-R	TACAGTGGAAGGTACACGGC
Mmu-LINC159-F	TGTCCACAGGAATGTGGCTT
Mmu-LINC159-R	TGTGTCAACTTTGGGAGGCT
Mmu-LINC153-F	TCATCAGGAGGCTGGATGGA
Mmu-LINC153-R	CTGGGAATGAGAGCTAGCGG
Mmu-Kcnq1ot1-F	ACAATTTCAAGCTCGGCGGT
Mmu-Kcnq1ot1-R	CTGCCTCTGCCTCTTGGC
Mmu-Runx1-F	GCGGTAGAGGTGCGTATCTG
Mmu-Runx1-R	TGCGTTCCAAGTCCATCACA
Mmu-B830012L14-F	GGTTCTGCCACGAGGATTCA
Mmu-B830012L14-R	TCACAGTACGGCTGCCATTT
Mmu-Gapdh-F	TCACCACCATGGAGAAGGC
Mmu-Gapdh-R	GCTAAGCAGTTGGTGGTGCA
Mmu-Alp-F	ATCTTTGGTCTGGCTCCCATG
Mmu-Alp-R	TTTCCCGTTCACCGTCCAC
Mmu-Bglap-F	CTGTCTCTCTGACCTCACA
Mmu-Bglap-R	GTGGGCTCCAGGGGATCT
Mmu-Col1-F	AGTCGCTTCACCTACAGCAC
Mmu-Col1-R	TTCGATGACTGTCTTGCCCC
Mmu-MENε/β-F	GTTTCCGGTCACCCAAATGC
Mmu-MENε/β-R	GCACTGCTACGACTCACACT
Mmu-MENβ-F	TTGTCAACTCTCTGGGAGGGA
Mmu-MENβ-R	CCCTTACCTGCAGTGCCATT
Mmu-NONO-F	TCCTCCTGCATTCAACCGTC
Mmu-NONO-R	GCCTTCAAGCATGCTCCCTA
Mmu-SFPQ-F	AATGGGTGGTGGTGGAACAA
Mmu-SFPQ-R	CCAAAGCGCTCAGTACGCAT
Mmu-Smurf1-F	AGCATCAAGATCCGTCTGACA
Mmu-Smurf1-R	CCAGAGCCGTCCACAACAAT
Mmu-Wwp1-F	TGGCTGCTTCTGTGCTCAAG

Mmu-Wwp1-R	GGGGTGGGGACATTCAAACA
Mmu-Smurf2-F	AAACAGTTGCTTGGGAAGTCA
Mmu-Smurf2-R	TGCTCAACACAGAAGGTATGGT
Mmu-Chip-F	CCTATGACCGCAAGGACATT
Mmu-Chip-R	CTCTACCCAGCCGTTCTCAG
Mmu-Nedd4-F	GCTTTTTCAACACCGCCGAT
Mmu-Nedd4-R	ATCTCCAGAGGGTTCCGACA
Mmu-actin-F	GTGACGTTGACATCCGTAAAGA
Mmu-actin-R	GCCGGACTCATCGTACTCC
Mmu-U1-F	GGCGAGGCTTATCCATTG
Mmu-U1-R	CCCACTACCACAAATTATGC
**Human**	
Primer	Sequence(5'to3')
hm-MENβ-F	AAACGCTGGGAGGGTACAAG
hm-MENβ-R	ATGCCCAAACTAGACCTGCC
hm-MENε/β-F	CTGAACTTAGCTCGACGGGG
hm-MENε/β-R	GCGCCTTAACTCCACATCAC
hm-Gapdh-F	CAATGACCCCTTCAT TGACC
hm-Gapdh-R	GACAAGCTTCCCGTTCTCAG
hm-ALP-F	GTGAACCGCAACTGGTACTC
hm-ALP-R	GAGCTGCGTAGCGATGTCC
